# Host habitat is the major determinant of the gut microbiome of fish

**DOI:** 10.1186/s40168-021-01113-x

**Published:** 2021-07-31

**Authors:** Pil Soo Kim, Na-Ri Shin, Jae-Bong Lee, Min-Soo Kim, Tae Woong Whon, Dong-Wook Hyun, Ji-Hyun Yun, Mi-Ja Jung, Joon Yong Kim, Jin-Woo Bae

**Affiliations:** 1 Department of Biology and Department of Life and Nanopharmaceutical Sciences, Kyung Hee University, Dongdaemun-gu, Seoul, 02447 Republic of Korea; 2 Biological Resource Center, Korea Research Institute of Bioscience and Biotechnology, Jeongeup, Jeollabuk-do 56212 Republic of Korea; 3 Distant-water Fisheries Resources Division, National Institute of Fisheries Science, Gijang-eup, Busan, 46083 Republic of Korea

**Keywords:** Fish, Fish gut microbiota, Freshwater fish, Seawater fish, Vertebrate gut microbiota

## Abstract

**Background:**

Our understanding of the gut microbiota of animals is largely based on studies of mammals. To better understand the evolutionary basis of symbiotic relationships between animal hosts and indigenous microbes, it is necessary to investigate the gut microbiota of non-mammalian vertebrate species. In particular, fish have the highest species diversity among groups of vertebrates, with approximately 33,000 species. In this study, we comprehensively characterized gut bacterial communities in fish.

**Results:**

We analyzed 227 individual fish representing 14 orders, 42 families, 79 genera, and 85 species. The fish gut microbiota was dominated by *Proteobacteria* (51.7%) and *Firmicutes* (13.5%), different from the dominant taxa reported in terrestrial vertebrates (*Firmicutes* and *Bacteroidetes*). The gut microbial community in fish was more strongly shaped by host habitat than by host taxonomy or trophic level. Using a machine learning approach trained on the microbial community composition or predicted functional profiles, we found that the host habitat exhibited the highest classification accuracy. Principal coordinate analysis revealed that the gut bacterial community of fish differs significantly from those of other vertebrate classes (reptiles, birds, and mammals).

**Conclusions:**

Collectively, these data provide a reference for future studies of the gut microbiome of aquatic animals as well as insights into the relationship between fish and their gut bacteria, including the key role of host habitat and the distinct compositions in comparison with those of mammals, reptiles, and birds.

**Video Abstract**

**Supplementary Information:**

The online version contains supplementary material available at 10.1186/s40168-021-01113-x.

## Background

Multicellular eukaryotes appeared 1.2 billion years ago, followed by a long evolutionary history of mutual interactions between multicellular and single-celled organisms [1]. According to Van Valen’s “Red Queen hypothesis,” evolution is driven by competition among taxa for survival under constantly changing environments [2]. Indeed, co-existence with microbes poses one of the greatest challenges for animals. At the same time, hosts and microbes can establish symbiotic relationships, in which each species benefits from mutualistic interactions [3]. The symbiotic microbiota contributes to animal adaptation to various habitats by providing complementary functional resources (e.g., by digesting indigestible dietary fiber, producing essential vitamins, protecting against enteropathogens, maintaining immune homeostasis, and contributing to intestinal maturation) over a long period of co-evolution [4–10].

In the last decade, numerous studies have explored gut microbial communities of various animal hosts. However, these studies have mostly focused on the gut microbiota of mammals [11], which represent less than 10% of all vertebrate diversity. By contrast, there are more than 33,000 species of fish, representing the greatest species diversity among groups of vertebrates [12, 13]. This focus on a single class of animals allows only limited insight into the vertebrate gut microbiota. To understand the co-evolution of vertebrates and gut microbes, broad analyses of fish are essential. The gut microbiota has been evaluated in a few model fish species, such as zebrafish [14], guppy [15], and rainbow trout [16], and in economically valuable aquatic animals, such as carp [17], Atlantic salmon [18], sturgeon [19], and Atlantic cod [20]. However, these studies are insufficient to comprehensively understand the composition of the gut microbiota in fish and patterns of co-evolution.

**Fig. 1:**
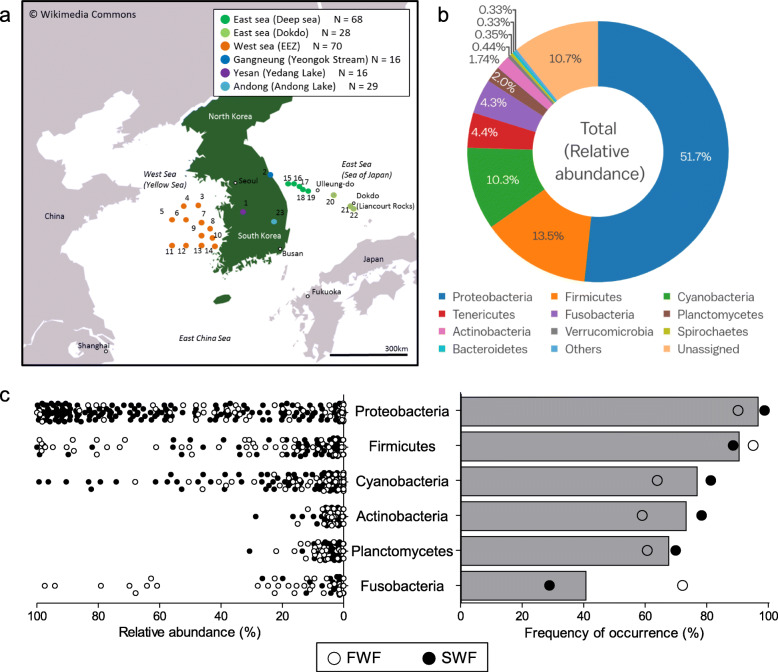
Overview of the data. **a** Regional map showing the approximate locations of 23 sampling sites (227 fish). **b** Pie chart of the relative abundance of bacterial phyla (> 0.3%) in the gut microbiota in all fish samples. **c** Dot plot of the overall distribution of the relative abundance (left) and frequency of occurrence (right) of taxa in total fish (bar) and freshwater fish or seawater fish (dot) at the bacterial phylum level. *FWF* freshwater fish, *SWF* seawater fish

**Fig. 2:**
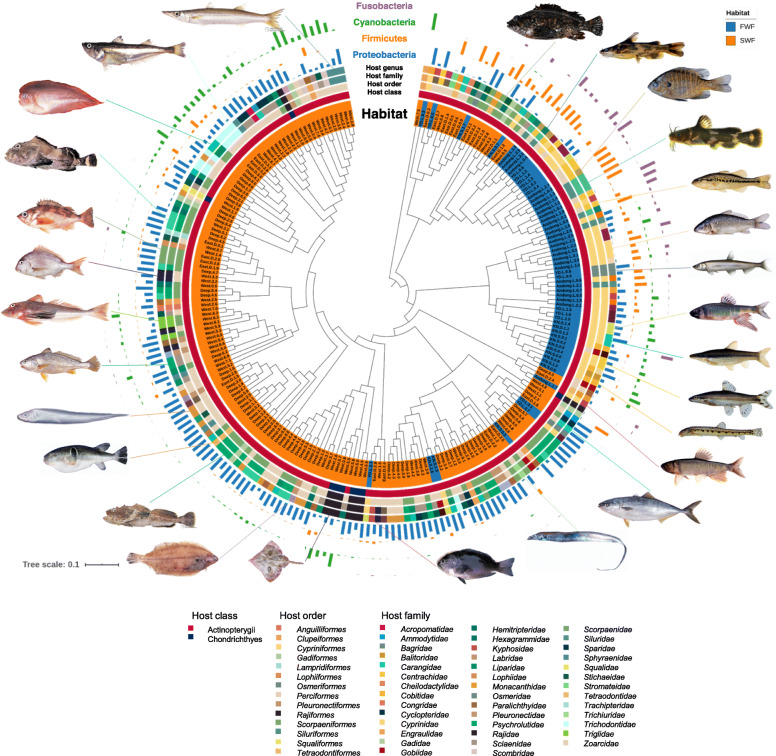
Overview of the gut microbiome of fish. UPGMA tree was constructed with 227 fish samples. Phylum-level profiling of the gut microbiome composition and host taxonomic attributes are presented with representative photos of fish species included in this study. Detailed color index of host genera was abridged. Fish photos courtesy of the National Institute of Fisheries Science (NIFS), Korea

Here, we aimed to resolve long-standing questions about the gut microbiota in fish. For example, is the gut microbiota in fish shaped by the host habitat? Do genetic factors in fish affect the structure of the gut microbiota and, if so, to what extent? How does the gut microbiota of fish differ from those of other vertebrates? To resolve these issues, we comprehensively characterized the gut microbiota of 227 individual fish representing 85 species obtained from lakes, a stream, and seas (i.e., habitats with distinct differences in nutrient availability, salinity, temperature, and depth) (Figs. 1 and 2). We used a clustering approach to find the primary determinants of the structures of the gut microbiome and verified these determinants using unsupervised and supervised machine learning approaches, likes PAM clustering and random forest classification. To gain a wider perspective, we compared gut microbial communities in fish and other vertebrates (mammals, reptiles, and birds) by using principal coordinate analysis (PCoA). These data serve as a reference for future studies of the gut microbiota of fish and other aquatic animals. Our findings also support the notion that symbiotic relationships between microbes and vertebrates are important for adaptation and provide insights into the nature of interspecific microbiome variation in various fish species.

## Results

### Overview of data related to the gut microbiota in fish taxa

We analyzed the bacterial community composition of the intestinal contents of 227 individual fish inhabiting six different environments (23 different sampling spots; Fig. 1a). The collected fish were taxonomically grouped into two classes (ray-finned and cartilaginous fish), 14 orders, 42 families, 79 genera, and 85 species. Overall, 1,014,240 raw 16S rRNA gene sequence reads were obtained from the intestinal contents of fish, and 653,281 high-quality sequence reads were obtained after the removal of low-quality and chimeric sequences. The high-quality sequences were clustered into 3273 operational taxonomic units (OTUs), with a mean number of OTUs per sample of 91 (± 6 SD), applying a threshold of 97% sequence identity.

To determine whether the sampling depth was sufficient to give an overview of the fish gut microbiota, rarefaction curves were generated for the number of OTUs per individual or species (Additional file 1: Supplementary Fig. S1). The cumulative number of identified OTUs reached a plateau at approximately 150 individuals or 60 fish species. This pattern was not affected by the fish habitat, indicating that the sampling depth was sufficient to capture the global bacterial diversity of the gut microbiota of wild fish.

The fish gut microbiota included 21 bacterial phyla, with three dominant phyla (*Proteobacteria*, *Firmicutes*, and *Cyanobacteria*) accounting for over 70% of all sequence reads (Fig. 1b and Additional file 2: Supplementary Table S1). Notably, the detailed microbial community composition of the fish gut differed considerably from the typical composition of the gut microbiota in vertebrates, mainly composed of *Firmicutes* and *Bacteroidetes* [21–23]. *Proteobacteria* was the most frequent taxon in the fish gut at the phylum level (detected in 219 fish samples), followed by *Firmicutes*, *Cyanobacteria*, and *Planctomycetes*. Although *Fusobacteria* was present in less than 50% of total samples, it was frequently detected in freshwater fish (Figs. 1c and 2).

### Host habitat is the major determinant of the gut microbiota of fish

Environmental factors and host genetics shape the gut microbiota of various animal taxa [21, 24, 25]. However, the extent to which these factors contribute to the microbiome composition of fish is unclear. To evaluate the relative importance of various factors, we first examined the similarity of the microbial community using within-sample distances for various clustering scenarios. We found significant variation at both within and between groups; however, the largest differences in microbial communities were obtained for factors related to the host habitat (salinity and sampling sites), and some groups even showed contrasting relationships with these factors (Additional file 1: Supplementary Fig. S2). These results indicate that environmental factors, particularly those associated with properties of the habitat, interact to shape the gut microbiota of fish.

**Fig. 3:**
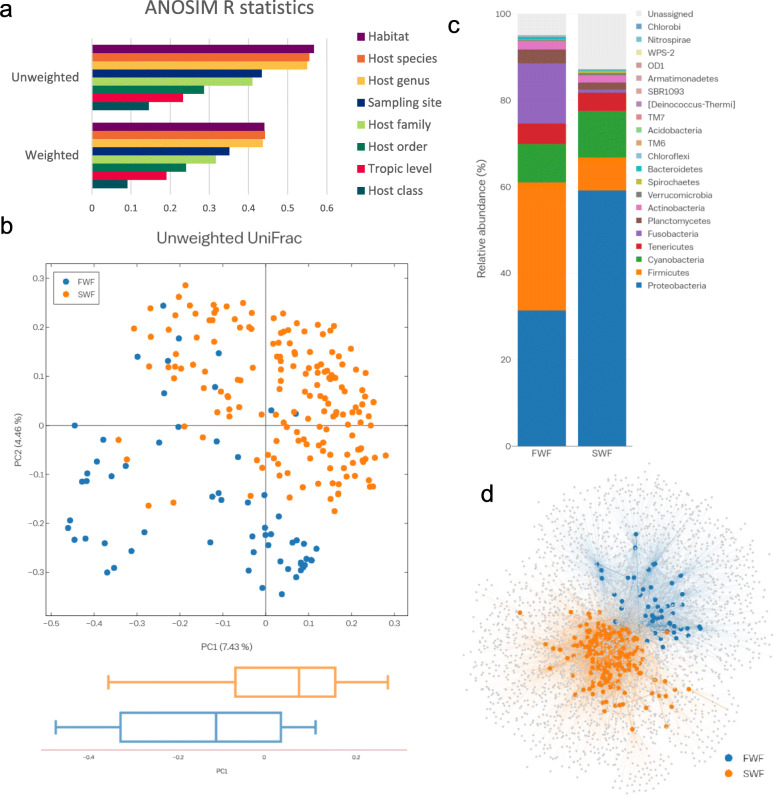
Fish gut microbiota is determined by the host habitat. **a** Analysis of the contributions of host environmental or genetic factors to the fish gut microbiota. Variation was determined by between-sample unweighted or weighted UniFrac distances. The size effect and statistical significance were calculated by ANOSIM using the R “vegan” package in the QIIME pipeline. **b** PCoA of unweighted UniFrac distances for 227 fish samples (ANOSIM, *R* = 0.47, *p* < 0.001) and boxplots illustrating PC1 coordinates of freshwater and seawater fish. The center line shows the median, the boxes cover the 25th to 75th percentiles, and the whiskers extend to 1.5× the interquartile range **c** Bar charts of the relative abundance of bacterial phyla in the gut microbiota of fish from different habitats. **d** OTU network-based analysis of the microbial communities in fish from different habitats. The edges connecting nodes representing fish samples (circles) to species-level OTUs in a particular sample are colored according to the host habitat type (edge-weighted spring embedded model in Cytoscape v. 3.0.1). *FWF* freshwater fish, *SWF* seawater fish

We next performed a clustering analysis using the partitioning around medoids (PAM) clustering algorithm based on the Calinski–Harabasz index and the silhouette score [26] to identify the optimal number of clusters and to evaluate the importance of environmental and genetic factors. The PAM clustering results showed that the gut microbiota of fish could be clustered into two groups, and the clusters were more consistent with variation in the host habitat (freshwater vs. seawater) than host class (Actinopterygii vs. Chondrichthyes) (Additional file 1: Supplementary Fig. S3a). To validate the importance of host habitat in shaping the gut microbiota of fish, we further assessed cluster validity for *k*-clusters, according to the following categories: habitat (number of variants, *n* = 2; freshwater vs. seawater), sampling site (*n* = 6; Fig. 1a), host order (*n* = 8), host family (*n* = 18), and host genus (*n* = 30). Among various categories, the habitat had the highest proportion of correctly matched constituents (Additional file 1: Supplementary Fig. S3b), indicating that habitat was the primary determinant of the fish gut microbiome. Compared with the former unsupervised learning approach (PAM clustering), we additionally evaluated associations between the various candidate factors and gut microbiota using the *R* statistic from analysis of similarities (ANOSIM) based on unweighted and weighted UniFrac distances. While all of the factors significantly (*p* < 0.001) affected the microbial structure of the fish gut, habitat and host species had the greatest ability to distinguish among samples (Fig. 3a).

We then performed a comparative analysis of habitat signatures in the gut microbiota of fish. With respect to α-diversity indices, the gut microbiota of freshwater fish exhibited significantly higher values for microbial richness (Shannon index), non-phylogenetic diversity (observed species), and equitability (Simpson evenness) than those of seawater fish, while Faith’s phylogenetic diversity was comparable across habitats (Additional file 1: Supplementary Fig. S4). As expected from the *R* values presented in Fig. 3a, a PCoA plot of unweighted UniFrac distance metrics revealed that the gut microbial communities of freshwater fish and seawater fish clustered separately (ANOSIM, *R* = 0.471, *p* < 0.001; Fig. 3b). Furthermore, distinct clustering of gut microbial communities was apparent when considering a more detailed habitat category, the sampling site, which reflects the type of freshwater and seawater (e.g., stream, lake, coast, or deep sea) and the geographical region (Additional file 1: Supplementary Fig. S5).

Next, we investigated differences in the composition of the gut microbiota with respect to the habitat. Indeed, a phylum-level difference between freshwater fish and seawater fish was detected (Figs. 2 and 3c). To determine whether the taxa with differences in abundance could serve as biologically relevant biomarkers for freshwater and seawater fish, we performed a linear discriminant analysis (LDA) effect size (LEfSe) analysis at all taxonomic levels. The phylum *Proteobacteria* was enriched in seawater fish with a relatively high LDA score (LDA > 3.8), while the phyla *Firmicutes* and *Fusobacteria* were significantly enriched in freshwater fish (Additional file 1: Supplementary Fig. S6a). At the family level, *Moraxellaceae*, *Vibrionaceae*, and *Enterobacteriaceae* (all in the class *Gammaproteobacteria*), and *Alcaligenaceae* (*Betaproteobacteria*) were significantly more abundant in seawater fish than in freshwater fish, whereas *Aeromonadaceae* (*Gammaproteobacteria*) was significantly more abundant in freshwater fish. The family *Clostridiaceae* (*Clostridia*) was more abundant in freshwater fish than in seawater fish, whereas *Leuconostocaceae* (*Bacilli*) was more abundant in seawater fish. Furthermore, most OTUs belonging to *Fusobacteriaceae*, showing a higher frequency in freshwater fish than in seawater fish, were assigned to *Cetobacterium* at the genus level (Additional file 1: Supplementary Fig. S6b and Additional file 2: Supplementary Table S1).

We then used a network-based approach to test whether gut microbial communities clustered by fish habitat at the OTU level. In the analysis, a node represents an individual fish and the OTUs are connected to the host fish in which they were detected. In agreement with the compositional differences noted above, in the OTU network-based analysis, the host nodes were more likely to connect to nodes of other hosts sharing the same habitat than to those from different habitats (Fig. 3d). Based on the OTU network-based analysis, we found that freshwater fish had a significantly higher number of connections (i.e., degree) and higher betweenness centrality than those of seawater fish, while seawater fish had higher neighborhood connectivity (Additional file 8: Supplementary Table S7). These results suggested that microbial diversity in freshwater fish is greater than that in seawater fish, analogous to the results based on alpha-diversity estimates.

In ecology, the trophic level is the position occupied by an organism in the food chain. Primary consumers, usually herbivores, occupy lower trophic levels, while predatory species (e.g., carnivores) occupy higher levels [27, 28]. Hence, we further investigated the trophic level of individual fish species to assess the effect of the host dietary gradient on the microbial community. A PCoA plot considering the trophic level, as determined using FishBase [13], revealed a distinct microbial gradient based on the ecological position of fish in the food chain (Additional file 1: Supplementary Fig. S7). These results suggested that the host trophic level is weakly but significantly (ANOSIM, *R* = 0.14, *p* < 0.001) associated with the gut microbial community assemblage.

### Host divergence had little influence on the gut microbiota of fish

We observed a statistically significant relationship between the gut microbial community structure and the host genetic variation in the cytochrome c subunit I (*CO1*) gene, although the degree of distinguishability did not exceed that for the habitat, as determined by the *R* value from ANOSIM. The host taxon-dependent variation in the gut microbial community was greater at lower taxonomic levels than at higher taxonomic levels (Fig. 3a). Remarkable variation in both the microbial community composition and structure was observed with respect to the host order (Additional file 1: Supplementary Fig. S8a). Differences in the relative abundances of several bacterial taxa depended on the host order. For example, *Epsilonproteobacteria* was relatively enriched in Tetraodontiformes, whereas the relative abundance of *Gammaproteobacteria* was higher in Lampriformes and Osmeriformes than in other host orders. *Firmicutes*, mainly represented by the class *Clostridia*, was relatively more abundant in Siluriformes, Gadiformes, Cypriniformes, and Osmeriformes than in other host orders. The phylum *Cyanobacteria* was enriched in Perciformes, Rajiformes, Clupeiformes, and Lophiiformes, whereas the phylum *Fusobacteria* was over-represented in Perciformes, Tetraodontiformes, Siluriformes, Cypriniformes, and Lophiiformes. Microbial communities showed significantly greater clustering within the same host order than across different host orders (ANOSIM, *R* = 0.20, *p* < 0.001) (Additional file 1: Supplementary Fig. S8b).

**Fig. 4:**
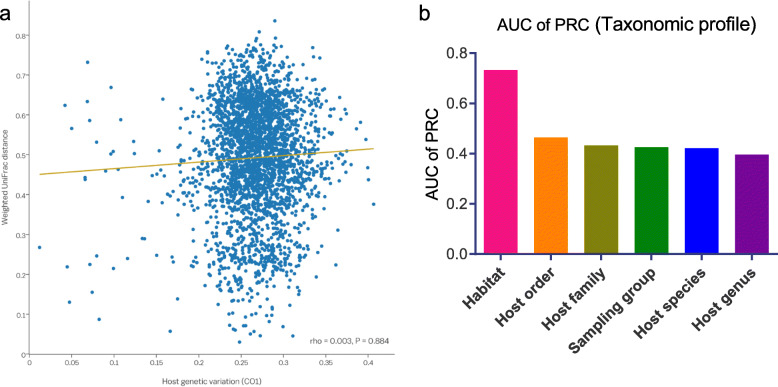
Limited evidence for an association between the fish gut microbiota and host genetic factors. **a** Pairwise comparison of phylogenetic distances between the fish gut microbiota based on weighted UniFrac distances and host genetic variation (CO1 gene). The relationship was not statistically significant (*p* = 0.884 and σ = 0.003, Spearman correlation). **b** AUC of PRC for random forest classifiers for various discriminative factors of the taxonomic profiles of the fish gut

Considering the host species-specificity of the gut microbiota in fish, we next investigated the existence of phylosymbiosis, or a relationship between host phylogeny and the gut microbiota. A scatter plot of weighted UniFrac distances plotted against host genetic relatedness based on variation in the *CO1* gene showed no significant association between similarity in the gut microbial community composition and host phylogenetic distance (Fig. 4a). Regardless of the phylogenetic distance among hosts, the dissimilarities of gut microbial communities between fish taxa were randomly distributed.

Habitat can be linked to host taxonomy because almost all fish inhabit specialized niches. To test whether this association confounds the distinguishability of the habitat/host taxonomy based on the gut microbiota, we next compared the microbial communities in Perciformes and Cypriniformes. In the current study, the order Perciformes was represented by 10 species (at least three individuals per species) caught in freshwater or seawater, and the order Cypriniformes included various freshwater species collected at multiple sampling sites (a stream and lakes). PCoA and UPGMA trees revealed that gut microbial communities from a single host order showed clearer clustering by habitat than by host species (Additional file 1: Supplementary Figs. S9 and S10), further supporting the greater role of the environment than host genetic factors in shaping the gut microbial community in fish.

In addition, we used a machine learning algorithm (the random forest classifier) to evaluate the predictive value of the microbial composition for host habitat or taxonomy. Owing to a large class imbalance in the number of variants per factor, precision-recall curves (PRC) were generated, and classification accuracy was calculated based on the area under the curve (AUC). The classification ability of the composition of the fish gut microbial community was better for discriminating the host habitat (freshwater vs. seawater) than for discriminating the sampling site or host taxonomy (Fig. 4b).

### Functional profiling of microbial communities

**Fig. 5:**
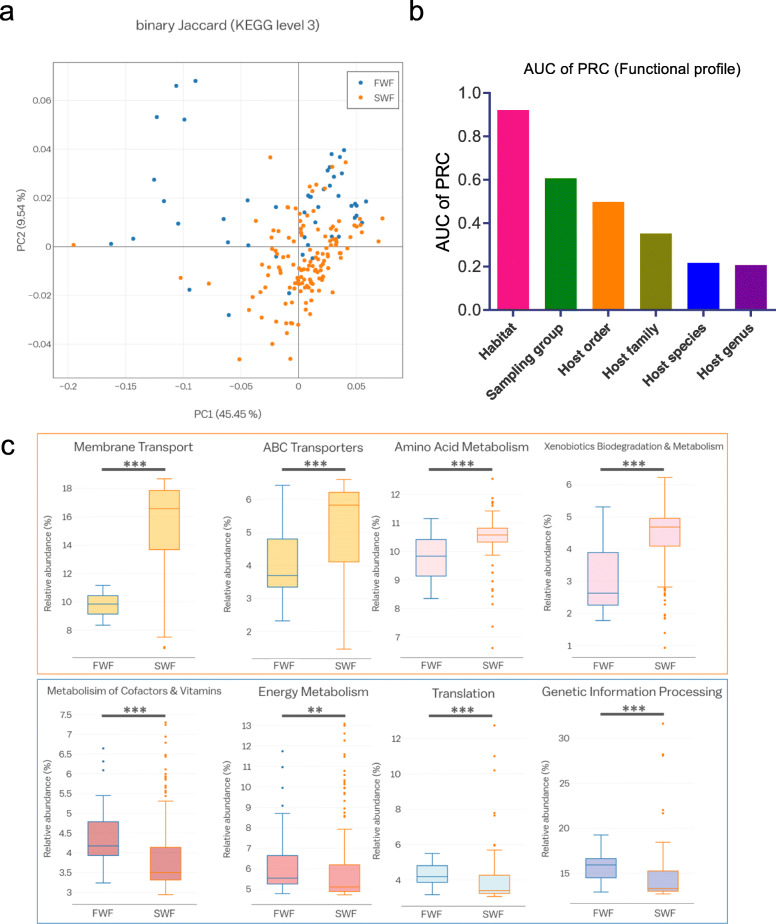
KEGG categories derived from the 16S rRNA sequences of the fish gut microbiome by PICRUSt. **a** PCoA of the binary Jaccard dissimilarity of the functional profiles (ANOSIM, *R* = 0.37, *p* < 0.001). **b** Box-and-whisker plots of the relative abundance of the selected KOs for samples from two different habitats determined by the LEfSe analysis (LDA score > 3.0). The center line shows the median, the boxes cover the 25th to 75th percentiles, the whiskers extend to 1.5× the interquartile range, and the outer points are outliers. Asterisks indicate significant differences between freshwater and seawater fish according to a two-tailed Mann–Whitney *U* test. **p < 0.01; ***p < 0.001. **c** Bar plot of the AUC of PRC for random forest classifiers for various discriminative factors of functional profiles of the fish gut. *FWF* freshwater fish, *SWF* seawater fish

We used the PICRUSt pipeline [29] to investigate whether the habitat-dependent differences in the microbial taxonomic composition are related to differences in functional profiles. Kyoto Encyclopedia of Genes and Genomes (KEGG) ortholog groups (KOs) predicted from the 16S rRNA gene sequences were assigned to broad functional categories based on the BRITE hierarchy. PCoA based on KOs predicted by PICRUSt revealed that the host habitat significantly affects the functional gene distribution (ANOSIM, *R* = 0.37, *p* < 0.001) (Fig. 5a). Most gene functions were related to metabolism (49.5%), environmental information processing (17.6%), and genetic information processing (14.5%) (Additional file 1: Supplementary Fig. S11 and Additional file 3: Supplementary Table S2). Gene families in the following categories were enriched in seawater fish: membrane transport, xenobiotic biodegradation and metabolism, amino acid metabolism, lipid metabolism, and transport and catabolism. By contrast, gene families in the following categories were enriched in freshwater fish: nucleotide metabolism, carbohydrate metabolism, metabolism of cofactors and vitamins, energy metabolism, translation, replication and repair, and cell motility (Fig. 5b).

We then used a machine learning approach to examine whether the functional profiles of the gut microbiota could be used to predict the environment or host taxon. The AUCs of PRC calculated using the functional profiles showed better prediction accuracy for the host habitat than for other factors, consistent with the results of the random forest classifier analysis based on microbial taxonomic profiles (Fig. 5c).

### Comparison of the gut microbiota of fish across geography and vertebrate clades

We evaluated the generalizability of our results for 85 fish species at a global scale by a comparative analysis with data for fishes caught in different regions (China, Saudi Arabia, Austria, and the USA) [30–34]. The first principal coordinate based on Bray–Curtis distances revealed that the gut microbial communities in this study had highest microbial diversity among reported gut microbiomes (Additional file 1: Supplementary Fig. S12).

**Fig. 6:**
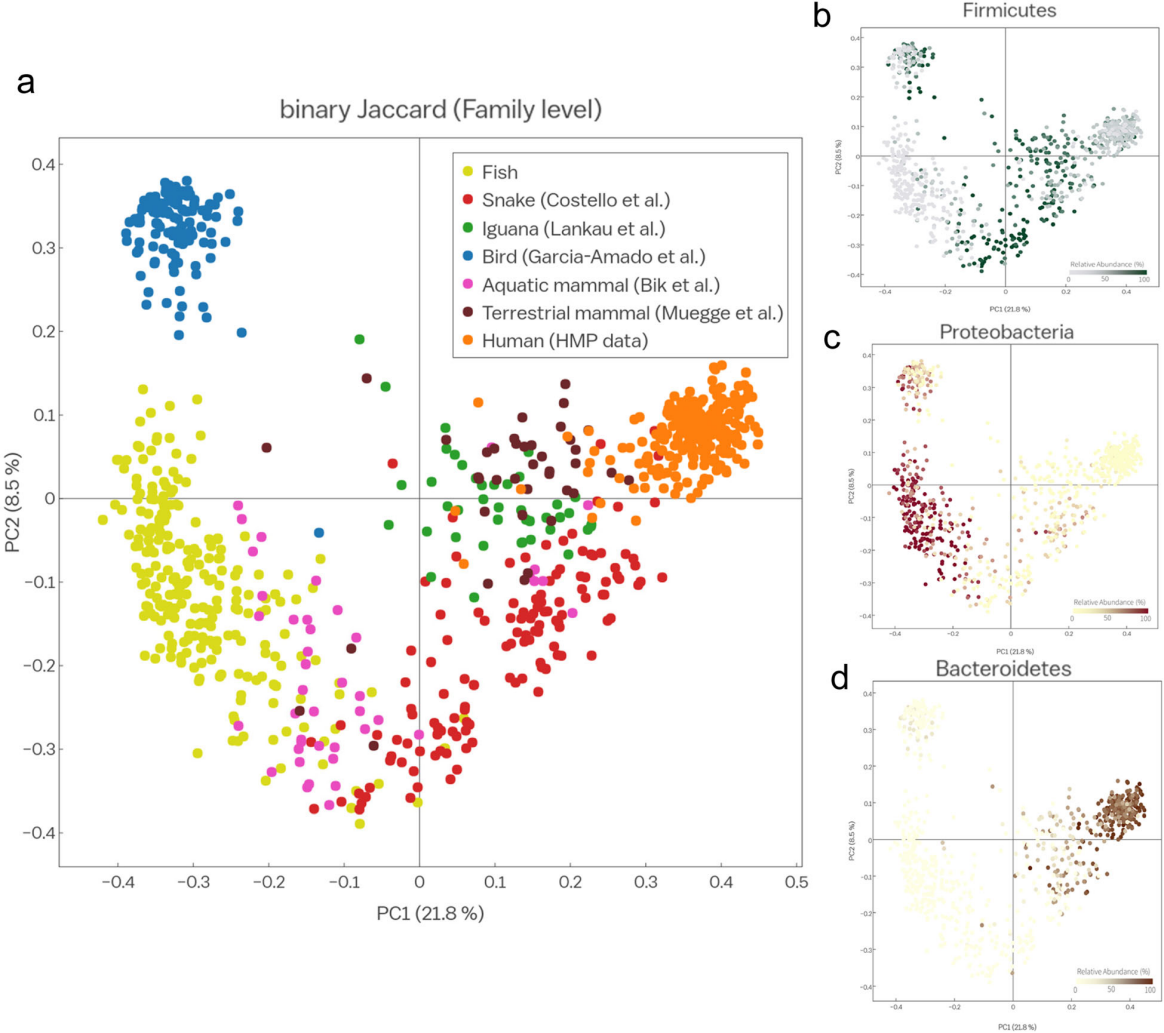
Fish gut microbiota clustered separately from the microbiotas of other vertebrates. **a** PCoA of the binary Jaccard indices of the gut microbiota from fish and various vertebrates (iguanas, snakes, aquatic mammals, birds, terrestrial mammals, and humans; sequences were obtained from the NCBI SRA and the Qiita server). **b**–**d** PCoA plots colored by the relative abundance of the phyla *Proteobacteria* (**b**), *Firmicutes* (**c**), and *Bacteroidetes* (**d**). Color intensity is proportional to the relative abundance (%) of each phylum

Lastly, we examined the impact of vertebrate evolution on gut microbes by comparing the microbial taxonomic profiles among fish and other vertebrate species. We compared the microbiota data for fish obtained in the current study with data for humans (Human Gut Microbiota Project [HMP] data [35, 36]), 66 aquatic mammals [37], 39 non-human mammals [38], 41 iguanas and snakes (reptiles) [39, 40], and 124 wild birds [41]. PCoA of the Bray–Curtis dissimilarity index and the binary Jaccard index revealed that the fish gut microbiome clustered separately from the other microbiomes (PERMANOVA, *p* < 0.001 and *p* < 0.001, respectively). Furthermore, the gut bacterial communities from other animals were clearly separated (Additional file 4: Supplementary Table S3). Clustering by phylogenetic relationships among hosts was clearly evident in a PCoA of the binary Jaccard index at the family level (Fig. 6), and significant differences were observed (ANOSIM, *R* = 0.64, *p* < 0.001). As shown in Fig. 6a, the gut microbiome of fish showed slight overlap with the gut microbiomes of aquatic mammals (dolphins and sea lions) and snakes, while the avian gut microbiome was distinct from those of other vertebrates. When examining the microbial composition of each host group, the components of the gut microbiota of each host differed at the microbial phylum level. *Proteobacteria* was most abundant in fish, *Firmicutes* was enriched in birds and reptiles, and *Bacteroidetes* was enriched in humans (Fig. 6b–d).

## Discussion

We characterized the gut microbial communities of various wild fish. Few studies have focused on the fish gut microbiota, despite the importance of fish in the evolutionary history of vertebrates and the tremendous species diversity, accounting for nearly half of all vertebrate species [13]. The gut microbiota of vertebrates is host-specific and arose as a result of co-evolution between hosts and microbes [42, 43]. Even in invertebrate species (e.g., shrimp or insect species), the gut microbiota is distinguished by the presence of specific commensal bacterial consortia [44, 45]. As expected, we found that the gut microbiota of wild fish is a host-specific and deterministic microbial assemblage. Furthermore, we showed that the gut microbiota is primarily determined by the fish environment, rather than by genetic factors.

The gut microbiota of most vertebrates, including amphibians, reptiles, mammals, and birds, is dominated by the phyla *Firmicutes* and *Bacteroidetes* [22, 36, 46, 47]. Indeed, a bloom of *Proteobacteria* is considered a sign of dysbiosis or instability of the gut microbial community in mammals [48]. Many commensal *Proteobacteria* can become pathobionts, infecting the host under specific conditions and facilitating inflammation [49, 50]. However, in the current study, *Proteobacteria* dominated the gut microbiota of the majority of fish, in agreement with recent studies of the gut microbiota of fish [14, 15]. These compositional differences at the phylum level can be explained by a partial projection of the vast diversity of marine *Proteobacteria* associated with the unsegmented digestive system of fish, unlike that of mammals [51, 52]. A stochastic assemblage of environmental microbes in the fish gut microbiota is unlikely because the predominant bacterial taxa in the ocean or other aquatic habitats, such as SAR11 (*Pelagibacter ubique* HTCC1062), SAR116 (*Puniceispirillum marinum* IMCC1322), and SAR86, were absent or not abundant in the fish gut (Additional file 5: Supplementary Table S4) [53, 54]. Our results were based on observations of both the gut digesta and mucosa of collected fish, which are essentially microbial reservoirs including allochthonous and autochthonous microbes, respectively. Nonetheless, predominant environmental microbes were rarely observed [55]. These findings prompt the question of whether *Proteobacteria* outcompete other environmental bacterial taxa in the aquatic habitat or whether they have been selected by the host itself [56–58].

We found that host habitat was the predominant determinant of the fish gut microbial community. Assessments of the discriminative structuring factors of the gut microbiota using both unsupervised and supervised learning approaches, such as PAM clustering, ANOSIM, and random forest classifier analysis, supported the importance of habitat, and this was particularly apparent in fish with a similar genetic background (e.g., Perciformes and Cypriniformes). Nevertheless, various other factors that were examined, including host taxonomy and trophic level, contributed to the fish gut microbial community (Fig. 3a). Environmental factors could not explain a large portion of the total variance (Fig. 3b; variation explained by PC1 at 7.43%) in the fish gut microbiota. Hence, intrinsic genetic factors are also important, and the species-specificity of the gut microbiota is a result of the intrinsic genetic background of the host.

Differences in the microbial compositional with respect to salinity can be explained in terms of host adaptation to the environment. The dominant taxa might reflect the affinity of the host for gut bacteria that contribute to the maintenance of immune function and metabolic activity. For example, the high proportion of *Fusobacteria* in freshwater fish might be associated with vitamin B_12_ (cobalamin). *Cetobacterium somerae* (order *Fusobacteriales*) is widely distributed in various freshwater fish, and its prevalence is negatively correlated with the dietary availability of vitamin B_12_ [59, 60]. Different environmental conditions affect vitamin B_12_ availability, and freshwater fish harbor more vitamin B_12_-synthesizing bacteria, such as *C*. *somerae*, to satisfy their dietary needs. The importance of metabolic properties is consistent with the predicted functions of gut bacteria in freshwater fish, which showed a relatively high abundance of genes related to the metabolism of cofactors and vitamins. This suggests that basic nutrient availability in the environment drives selection of the fish gut microbiota to account for the nutritional deficiencies of the host. Based on the performance of classifiers, better results are obtained when using functional profiles as a training trait than when using taxonomic profiles. Environmental factors (e.g., habitat type) result in functional redundancy, with the host physiology governed by the ability to adapt.

We also observed high similarity between the gut microbiota of hosts that share feeding preferences. The average trophic level of seawater fish collected in the current study was higher than that of freshwater fish. Seawater fish show carnivorous and herbivorous dietary preferences, while freshwater fish tend to show omnivorous dietary preferences [28]. In particular, the family *Enterobacteriaceae* was significantly enriched in seawater fish, consistent with results for other carnivorous fish [15, 61]. Further, a bloom of marine-associated bacteria, such as *Enterobacteriaceae* and *Moraxellaceae*, is correlated with a low-fiber or animal-based diet in humans [62, 63]. By contrast, *Clostridium* and *Aeromonadaceae* were predominant in freshwater fish in the current study. Several *Clostridium* species are well-known cellulose-degraders associated with herbivorous vertebrates [64, 65]. *Aeromonas* is dominant in fish feeding on detritus of plant origin and in omnivorous freshwater fish (intermediate trophic level) [15, 66]. Differences in the gut microbiota are not simply a consequence of the host diet or feeding preference, as divergence between the gut microbiota of freshwater and seawater fish can also be a cause of the functional potential of hosts (Fig. 5).

In a comparison between fish and other vertebrates, including Reptilia, Avia, and Mammalia, we detected clearly distinct structures of each gut microbiota (Fig. 6). This was observed despite analogous taxon with similar metabolic or biological roles, i.e., a relatively high proportion of *Enterobacteriaceae* and *Moraxellaceae* (*Proteobacteria*) in animal-based diet vertebrates [62, 63] and the dominance of *Clostridium* species (*Firmicutes*) in plant-based diet vertebrates [64, 65]. Unlike the gut microbial composition of fish, the dominant gut bacteria of terrestrial mammals and humans belong to the phyla *Firmicutes* and *Bacteroidetes*. *Firmicutes* is the sole prominent microbial phylum in the guts of reptiles and birds. This difference at the microbial phylum level can be explained by evolved differences between fish and other vertebrates in the selectivity of the gut environment [15, 67]. Early fish arose 600 million years ago and became ancestors of all extant vertebrate clades [12]. Since the appearance of early vertebrates, they have evolved a number of physiological adaptations for survival in various environments. During this process, symbiotic gut microbes and host species co-evolved to survive in the continuously changing environment. It is difficult to experimentally simulate gut microbial selection and colonization during vertebrate evolution; however, surveys and experiments involving extant vertebrate species can provide insight into the contribution of various environmental and genetic factors to the gut microbiota.

Our species-wide study included an unprecedented number of fishes; however, it had several limitations. Since sample collection focused on East Asia (the Korean peninsula), the taxa are not representative of the total species diversity of fish. Although the number of samples in our study was sufficient for capturing microbial diversity reported in various fishes from other regions (China, Saudi Arabia, Austria, and the USA), our findings may not be representative of all fish species. Further studies including a broader range of species or more detailed metadata for the surrounding environment (e.g., precise estimates of salinity, temperature, or prey composition) are necessary to elucidate the contributions of particular environmental factors to shaping the fish gut microbiota. The detailed characterization of ecological niches and metabolic differences among fish will improve our understanding of the fundamental assemblage of the gut microbial consortium in fish. Furthermore, we analyzed the 16S rRNA gene to evaluate the bacterial composition and predicted functional profiles using the PICRUSt pipeline. These analyses indicated that some taxa are linked to specific biological activities of the fish host. Additional meta-omics analyses, including shotgun metagenomic sequencing and metaproteomics and metabolomics approaches, could yield a more comprehensive dataset for detailed analyses of the determinants of the specific consortia of gut microbes in fish and expand our understanding of fundamental contributions of microbes to fish biology [68].

## Conclusions

In summary, our results provide a comprehensive view of the fish gut microbiota. In particular, we found that host habitat (freshwater vs. seawater) has a dominant role in shaping wild fish gut microbial communities over host taxonomy and trophic level. Moreover, the microbial functional profiles predicted from 16S rRNA gene sequences were predominantly determined by host habitat. We further demonstrate that random forest classifiers trained on microbial community composition or functional features showed better prediction accuracy for the host habitat than for other factors. In addition, the fish gut microbiome in a PCoA plot clustered separately from those of other vertebrates, such as mammals, reptiles, and birds. Our findings improve our understanding of the long-term co-evolution of vertebrates and their indigenous microbial communities.

## Methods

### Sample collection

Gut samples from 227 seawater and freshwater fish were collected at 23 sites in Korea between June 2013 and October 2013 (Figs. 1a and 2 and Additional files 6 and 7: Supplementary Tables S5-6). Seawater fish were caught by the fisheries resource research vessel Tamgu-20 of the National Institute of Fisheries Science (NIFS), Korea. During a seasonal fisheries resource investigation of the deep sea of East Sea, near the seas of Ulleung-do and Dok-do, and West Sea, 175 seawater fish were caught by bottom trawling, mid-water (pelagic) trawling, and trammel. Freshwater fish were collected in collaboration with the Inland Fisheries Research Institute (NIFS) by using cast net and fish traps. All procedures for the collection and handling of seawater and freshwater fishes were approved by the NIFS and performed under the supervision of authorized and experienced members of the NIFS staff. The seawater fish were handled in a laboratory facility on the fishing vessel and the freshwater fish were handled at appropriate facilities near the fishing sites. All fish were stunned and dissected immediately after catching. Approximately 1.0–1.5 cm of the rectum was collected using sterile instruments, and the samples, including the luminal content and mucosa, were stored at − 80 °C until analyses. An accompanying fish taxonomist identified the fish host species by briefly assessing fish morphological characteristics during sample collection. The fish host species were re-identified in the laboratory by a molecular phylogenetic analysis (*vide infra*).

### DNA extraction and pyrosequencing of bacterial 16S rRNA genes

The gut specimens were squeezed out with sterile instruments to collect the luminal content. The gut samples were cut laterally to remove the mucus layer of the fish gut by visual inspection. A cover glass was used to separate the mucus layer from the gut samples. The luminal content and mucus layer were pooled and transferred to a sterile conical tube containing 6.5 mM dithiothreitol for mucus degradation [69]. After incubation for 1 h at 37 °C, the pellet was collected by centrifugation and re-suspended in 750 μl lysis buffer (500 mM NaCl, 50 mM Tris-HCl, pH 8.0, 50 mM EDTA, and 4% sodium dodecyl sulfate). To maximize microbial cell lysis before DNA extraction, the re-suspended pellets were homogenized by shaking in a sterile screw-cap tube containing zirconia beads (2.3 and 0.1 mm diameter) and glass beads (0.5 mm diameter) using FastPrep-24 (MP Biomedicals, Santa Ana, CA, USA) for 50 s at 6.0 m/s. Genomic DNA from the homogenized samples was then extracted by the standard phenol-chloroform extraction method using the UltraClean Microbial DNA Isolation Kit (MOBIO, London, UK). The hypervariable regions V1–V3 of the 16S rRNA gene were amplified from the extracted genomic DNA of the sampled fish guts by using a sample-specific barcoded bacterial primer set [70] and Ex-Taq premix (Takara Bio, Kyoto, Japan). The polymerase chain reaction (PCR) conditions were as follows: 94 °C for 10 min; followed by 29 cycles of 94 °C for 60 s, 50 °C for 30 s, and 72 °C for 1 min 30 s; followed by a final extension step at 72 °C for 10 min. Four independent PCR products for each sample were pooled and purified using the QIAquick PCR Purification Kit (QIAGEN, Hilden, Germany). The concentration of purified PCR products was determined using the Quant-it PicoGreen dsDNA Assay Kit (Life Technologies, Carlsbad, CA, USA). The quality and quantity of DNA were checked using a Bioanalyzer 2100 instrument (Agilent, Santa Clara, CA, USA) and a DNA 1000 Lab Chip (Agilent). The pooled DNA was then amplified by emulsion PCR before 454 pyrosequencing using a GS FLX Titanium instrument (Roche, Basel, Switzerland) by a certified service provider (Macrogen, Seoul, Korea), according to the manufacturer’s instructions.

### Identification of fish host species and phylogenetic analysis of fish

To identify the fish host species, genomic DNA was extracted from the fish flesh collected aseptically from each specimen. Tissue fragments were suspended in 750 μl lysis buffer and homogenized by using FastPrep-24 (MPbio) with glass beads (0.5 mm diameter) for 40 s at 6.0 m/s. DNA was extracted using a standard phenol-chloroform extraction method. The *CO1* gene was amplified by using AccuPower PCR Premix (Bioneer, Daejeon, Korea) and the *CO1* gene primer cocktail set 3 [71]*.* The PCR conditions were as follows: initial denaturation at 95 °C for 3 min; followed by 30 cycles of 94 °C for 30 s, 52 °C for 40 s, and 72 °C for 1 min; followed by a final extension step at 72 °C for 10 min [71]. The PCR products were sequenced using the BigDye Terminator Cycle Sequencing Ready Reaction Kit (Applied Biosystems, Foster City, CA, USA), according to the manufacturer’s instructions. The reaction products were analyzed using an automated DNA analyzer system (PRISM 3730XL DNA Analyzer, Applied Biosystems). Sequence fragments were assembled using SeqMan (DNASTAR, Madison, WI, USA). The assembled *CO1* gene sequences were then compared with other *CO1* gene sequences in the nucleotide collection (nr/nt) of the GenBank database by searches using the Basic Local Alignment Search Tool (BLAST) [72]. The *CO1* gene sequences were aligned using the multiple alignment program CLUSTAL W (v. 1.4), and a phylogenetic tree was generated by using MEGA 6 [73, 74] using the maximum-likelihood algorithm with 1000 bootstrap replicates [75].

### Sequence analysis

The raw 16S rRNA sequences generated using the GS FLX Titanium platform were processed using QIIME (v. 1.8.0) [76]. All raw sequences with average quality scores below 25 and those shorter than 200 bp or longer than 1000 bp were removed. The quality-filtered sequences were denoised using the QIIME denoising algorithms [77]. The sequences were then clustered into OTUs at a 97% sequence similarity threshold using UCLUST [78] in QIIME. The OTUs were generated by searches against the Greengenes reference database from August 2013 using a subsampled open-reference method [79]. Before further analysis, chimeric sequences were detected by comparing with a reference database using USEARCH (v. 7.0.1090) [78] and were removed. A representative sequence for each OTU was picked and aligned with the Greengenes reference database by using PyNAST [80]. The alignments were used for phylogenetic tree construction using the FastTree algorithm [81]. An even-depth rarefied OTU table matrix (600 sequences) was constructed to calculate various diversity indices [82]. The Ribosomal Database Project classifier against the Greengenes reference database was used at a minimal confidence of 60% [83] for the taxonomic assignment of representative OTUs. The calculation of α-diversity indices (phylogenetic diversity, observed species count, Chao1 richness estimators, and the Shannon and Simpson indices) and β-diversity indices (Bray–Curtis dissimilarity and UniFrac weighted and unweighted metrics), as well as PCoA, were performed using QIIME pipelines. The calculated coordination was visualized using a web-based visualization tool, Plotly (http://plot.ly). To check for the presence of transient environmental bacteria in the gut microbiota, the full dataset was BLAST-searched against SAR11 (GenBank accession no. CP000084), SAR86 (JX530677), and SAR116 (CP001751) sequences. PICRUSt (http://picrust.github.io) [29] was used to examine the functional profiles of the fish gut microbial community based on the 16S rRNA gene composition. For the PICRUSt analysis, an OTU table was constructed by closed-reference OTU picking against the May 2013 Greengenes database using QIIME. The OTU table was converted into the PICRUSt format and normalized by the 16S rRNA gene copy number to correct for the over- and under-estimation of microbial abundance. The normalized dataset was analyzed using the KO dataset [84]. Detailed microbiome analytical scripts and computational environments are provided online (Additional file 10: Supplementary Method).

### Comparison of the gut microbiota among various animals

Gut microbiota data from various organisms were used for meta-analysis. For comparison with previously reported fish microbiome studies, we obtained the unprocessed microbiome sequence data from NCBI Sequence Read Archive (SRA) [30–34] (Additional file 9: Supplementary Table S8). All fish microbiome data were processed by QIIME pipeline and OTUs were clustered against Greengenes DB (ver. gg_13_8) with open-reference OTU picking methods (*pick_open_reference_otus.py*). Owing to differences in DNA extraction methods and sequencing platforms among studies, the constructed OTU table was collapsed to the genus-level and used for further analyses. The human gut microbiota dataset was downloaded from the NIH HMP (http://hmpdacc.org/) [36]. The aquatic mammalian gut microbiota [37] data were obtained from the NCBI SRA. The land and marine iguana gut microbiota data [39] were downloaded from the Dryad data package [85]. Non-human mammalian gut microbiota [38], snake gut microbiota [40], and wild avian gut microbiota [41] data were obtained from the Qiita database (https://qiita.ucsd.edu/), as pre-processed data. Closed-reference OTU picking methods (*pick_closed_reference_otus.py*) were used to cluster the OTUs against the same reference sequences (gg_13_8) using the QIIME pipeline (v. 1.8.0). After discarding the unaligned sequences, an even-depth rarefied OTU table was generated and used for subsequent analyses. A non-phylogenetic β-diversity metric (the binary Jaccard index) was calculated and visualized by PCoA.

### OTU network-based analysis

For an OTU network-based analysis, OTU network maps were constructed using QIIME and visualized using Cytoscape (v. 3.0.1) [86, 87]. Briefly, the OTU table generated at the 97% sequence similarity threshold was converted to the Cytoscape format (*make_otu_network.py*). In the converted OTU network maps, the samples and OTUs were set to represent network nodes connected by edges, which represented OTU abundance in the samples. The edge-weighted spring embedded model was derived to arrange network constituents. Topological analysis of OTU network was performed using Cytoscape and MCODE plug-in toolkit [88].

### Statistical analysis

All statistical analyses were performed using GraphPad Prism (v. 5.0; GraphPad, San Diego, CA, USA). The significance of differences between groups was assessed using two-tailed Mann–Whitney *U* tests. To compare the β-diversity indices among multiple groups, one-way analysis of variance was used, followed by Duncan’s post-hoc tests. For multiple comparisons, *p* values were corrected by the Benjamini–Hochberg false discovery rate (FDR) procedure, and FDR < 0.05 was considered statistically significant. ANOSIM and PERMANOVA tests with the β-diversity matrix were performed using the QIIME pipeline (*compare_categories.py*). Statistical significance for both tests was determined based on 10,000 permutations. Assessment models to identify the discriminative factors shaping the fish gut microbiota were constructed using random forest classifiers in Weka v. 3.8.3 open source software (http://www.cs.waikato.ac.nz/~ml/weka/index.html) developed at Waikato University, New Zealand [89, 90]. The random forest classifiers were trained using individually generated input tables of the relative OTU abundance and discriminative variables with 10-fold cross-validation. To determine the optimal number of clusters for evaluating the cohesiveness of clusters with various metadata, the Calinski–Harabasz index (CH index) and the silhouette score were calculated for each set of clusters generated by PAM clustering [26] (https://enterotype.embl.de/enterotypes.html#). The differentially abundant taxonomic and functional features were also confirmed using LEfSe in the Galaxy server (http://huttenhower.sph.harvard.edu/galaxy/) [91, 92]. The significance threshold of the α parameter for the Kruskal–Wallis test for classes was set to 0.05. The threshold for the logarithmic LDA score for taxonomic features was 3.8, and that for functional features was 3.0.

## Supplementary Information

**Additional file 1.** Supplementary Figures (S1–S12).

**Additional file 2.** Supplementary Table S1.

**Additional file 3.** Supplementary Table S2.

**Additional file 4.** Supplementary Table S3.

**Additional file 5.** Supplementary Table S4.

**Additional file 6.** Supplementary Table S5.

**Additional file 7.** Supplementary Table S6.

**Additional file 8.** Supplementary Table S7.

**Additional file 9.** Supplementary Table S8.

**Additional file 10.** Supplementary Method.

## Data Availability

The obtained 16S rRNA gene sequences for the fish gut microbiota and the CO1 gene sequences for collected fish were submitted to the European Nucleotide Archive (ENA) of EMBL-EBI and NCBI GenBank databases under the accession numbers PRJEB31232 (16S rRNA gene sequences) and MK560532-MK560758 (CO1 gene sequences), respectively.

## Abbreviations

ANOSIM: Analysis of similarities
AUC: Area under the curve
BLAST: Basic Local Alignment Search Tool
CO1: Cytochrome c oxidase subunit I
FDR: False discovery rate
KEGG: Kyoto Encyclopedia of Genes and Genomes
KO: KEGG orthology
LDA: Linear discriminant analysis
LEfSe: Linear discriminant analysis effect size
OTU: Operational taxonomic unit
PCoA: Principal coordinate analysis
PERMANOVA: Permutational multivariate analysis of variance
PICRUSt: Phylogenetic Investigation of Communities by Reconstruction of Unobserved States
PRC: Precision-recall curves
