# Supplementary material for: Host habitat is the major determinant of the gut microbiome of fish
Source: Microbiome. 2021 Jul 31;9:166. doi: 10.1186/s40168-021-01113-x (PMC8325807; doi:10.1186/s40168-021-01113-x)

Supplementary Figures for

**Host habitat is the major determinant of the gut microbiome of fish**

Pil Soo Kim^1†^, Na-Ri Shin^1†^, Jae-Bong Lee^2†^, Min-Soo Kim^1^, Tae Woong Whon^1^, Dong-Wook Hyun^1^, Ji-Hyun Yun^1^, Mi-Ja Jung^1^, Joon Yong Kim^1^ and Jin-Woo Bae^1*^

^1^Department of Biology and Department of Life and Nanopharmaceutical Sciences, Kyung Hee University, 26 Kyungheedae-ro, Dongdaemun-gu, Seoul 02447, Republic of Korea

^2^Distant-water Fisheries Resources Division, National Institute of Fisheries Science, Busan 46083, Republic of Korea

^†^These authors contributed equally to this work.

^*^Correspondence: baejw@khu.ac.kr


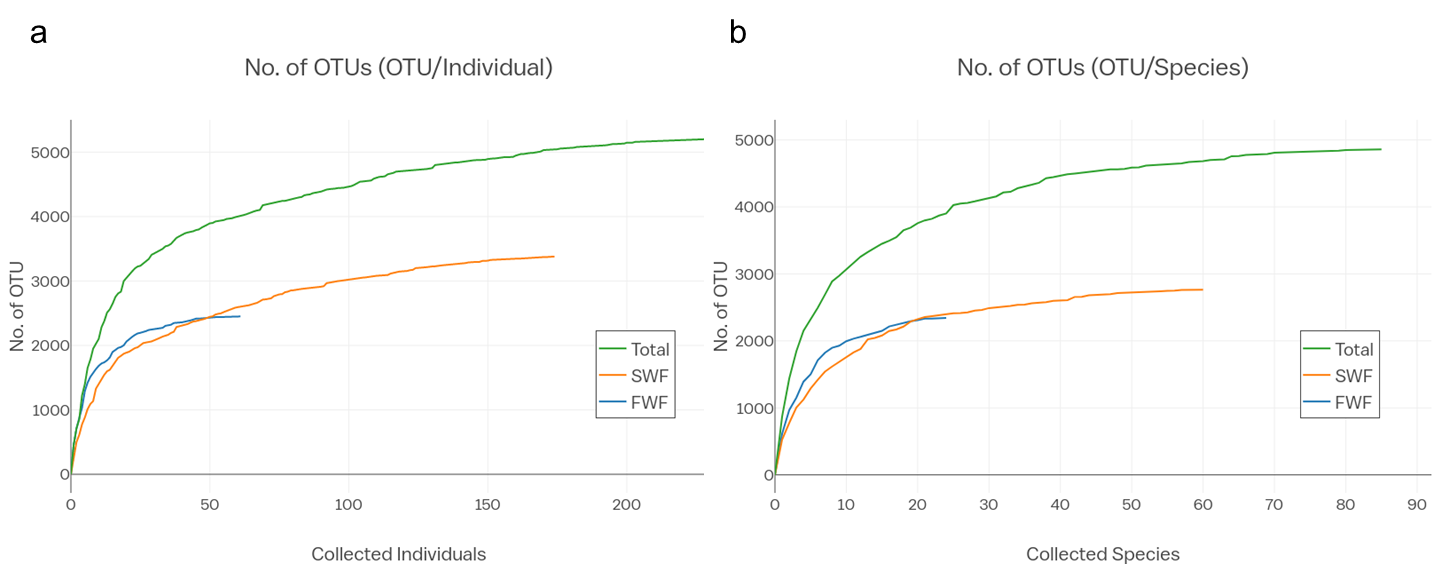


**Supplementary Fig. S1.** Rarefaction curves of the abundance-based coverage estimation against the cumulative number of identified OTUs. Coverage plots are shown for the individual samples (a) and species (b). FWF, freshwater fish; SWF, seawater fish.

**Supplementary Fig. S2.** Comparisons of beta diversity in the fish gut microbiome within groups and between groups, based on discriminating environmental and genetic factors. Asterisks indicate significant differences within and between groups according to a two-tailed Mann–Whitney *U*-test. *p < 0.05; **p < 0.01; ***p < 0.001.

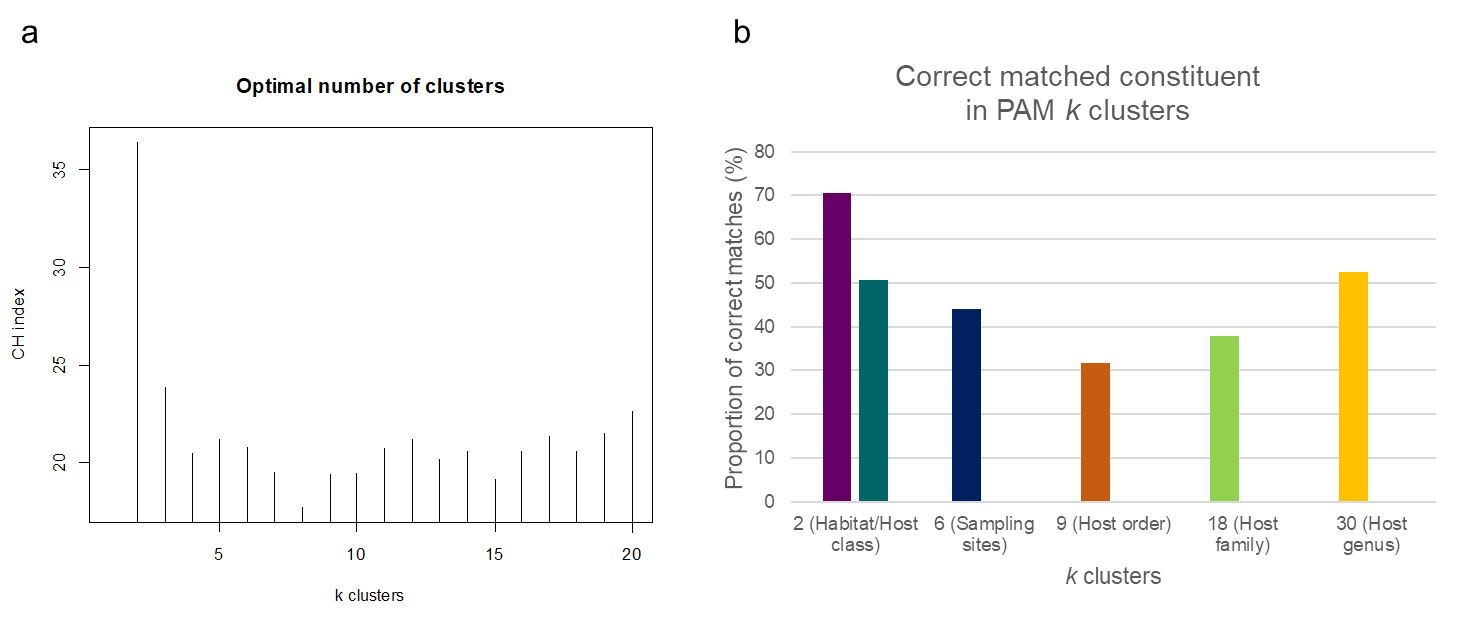


**Supplementary Fig. S3.** Identification of environmental and genetic factors shaping the fish gut microbiota by a clustering analysis. The partitioning around medoids (PAM) clustering algorithm based on the Calinski–Harabasz (CH) index was used. (a) The optimal number of enterotypes was two, as indicated by the maximum CH index. (b) The proportion of correctly matched constituents on PAM *k*-clusters based on various environmental and genetic factors.


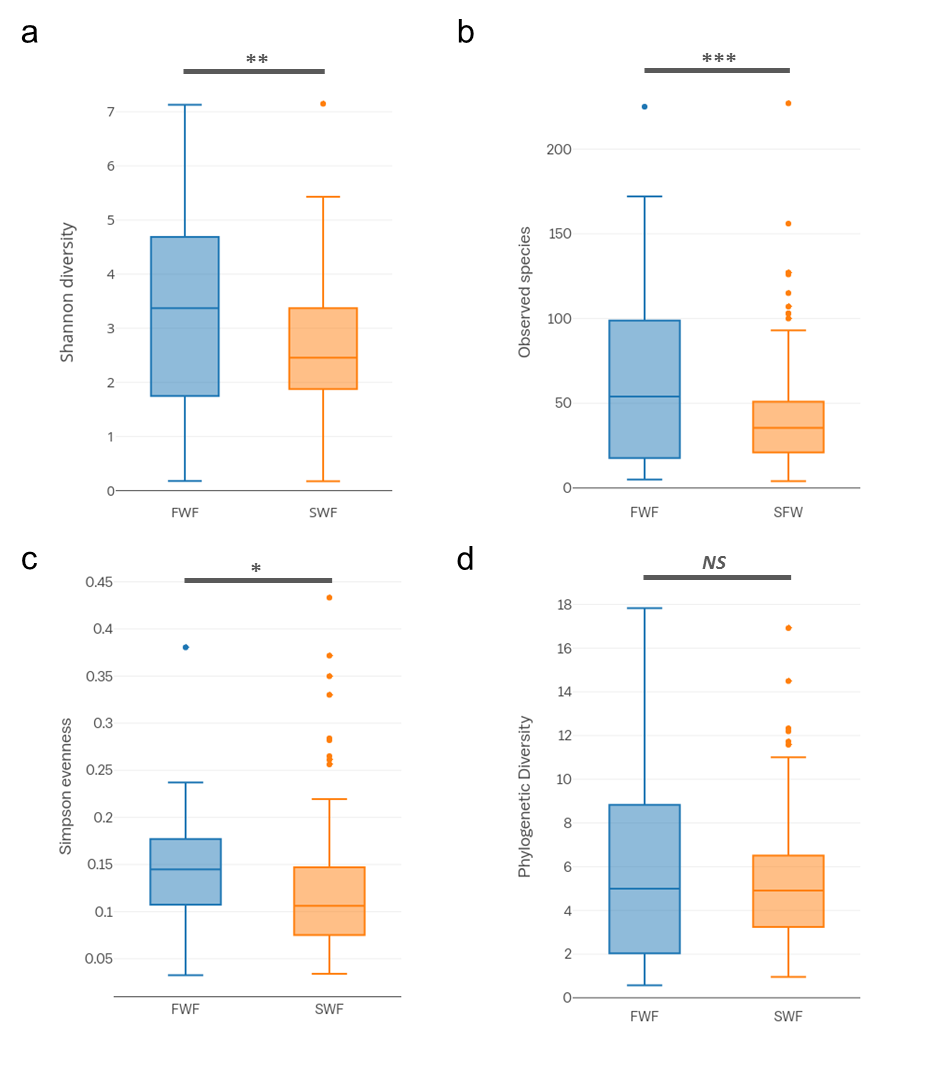


**Supplementary Fig. S4.** Alpha diversity indices of freshwater and seawater fish. (a) Shannon diversity. (b) Number of observed species. (c) Simpson evenness. (d) Faith’s phylogenetic diversity. The center line shows the median, the boxes cover the 25th to 75th percentiles, the whiskers extend to 1.5× the interquartile range, and the outer points are outliers. Asterisks indicate significant differences between the freshwater and seawater fish according to a two-tailed Mann–Whitney *U*-test with Benjamini–Hochberg FDR correction. *p < 0.05; **p < 0.01; ***p < 0.001; NS, not significant. FWF, freshwater fish; SWF, seawater fish.


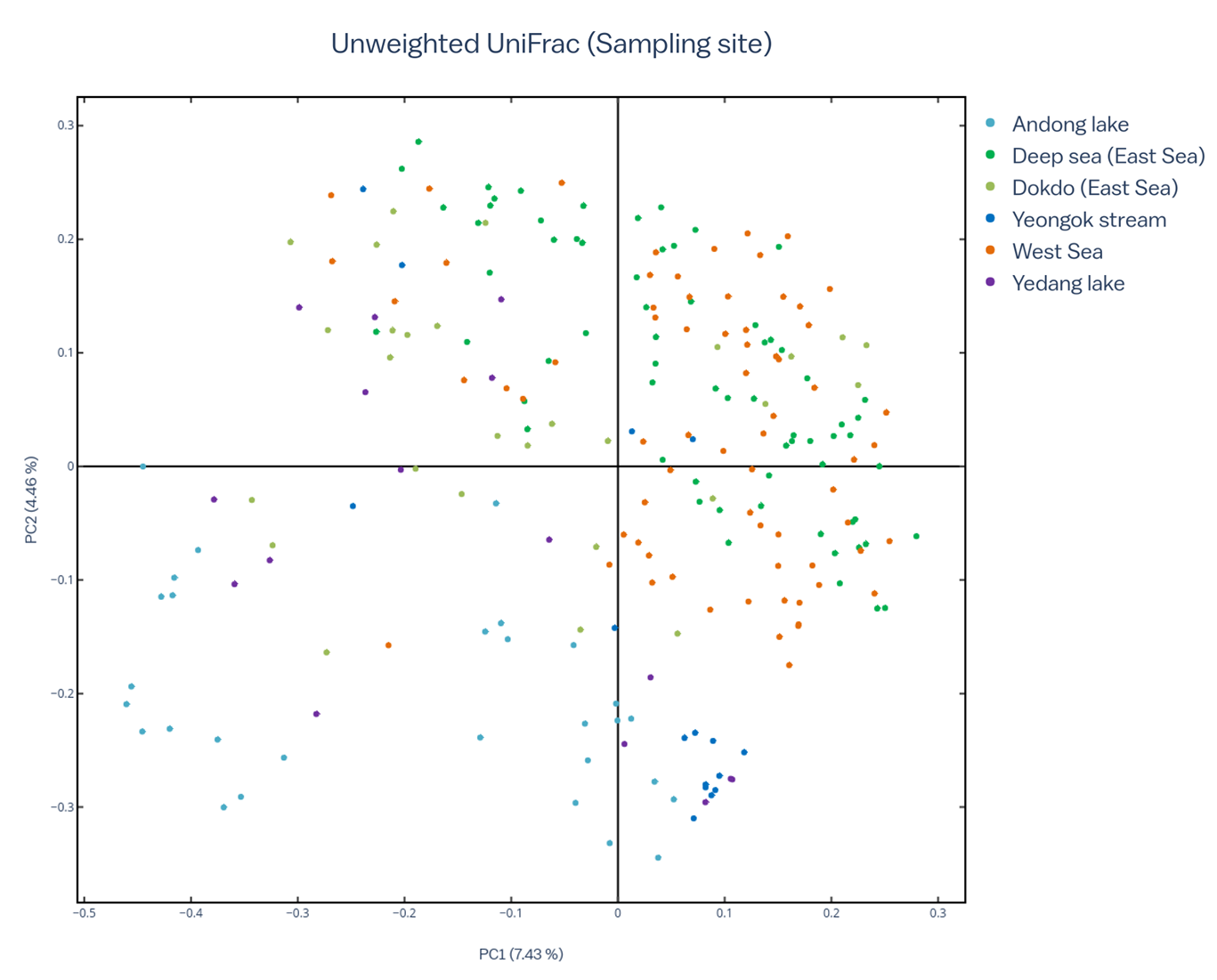
**Supplementary Fig. S5.** PCoA based on unweighted UniFrac distances for 227 fish gut microbiotas. Colors indicate collection sites (ANOSIM, *R* = 0.43, p < 0.001).


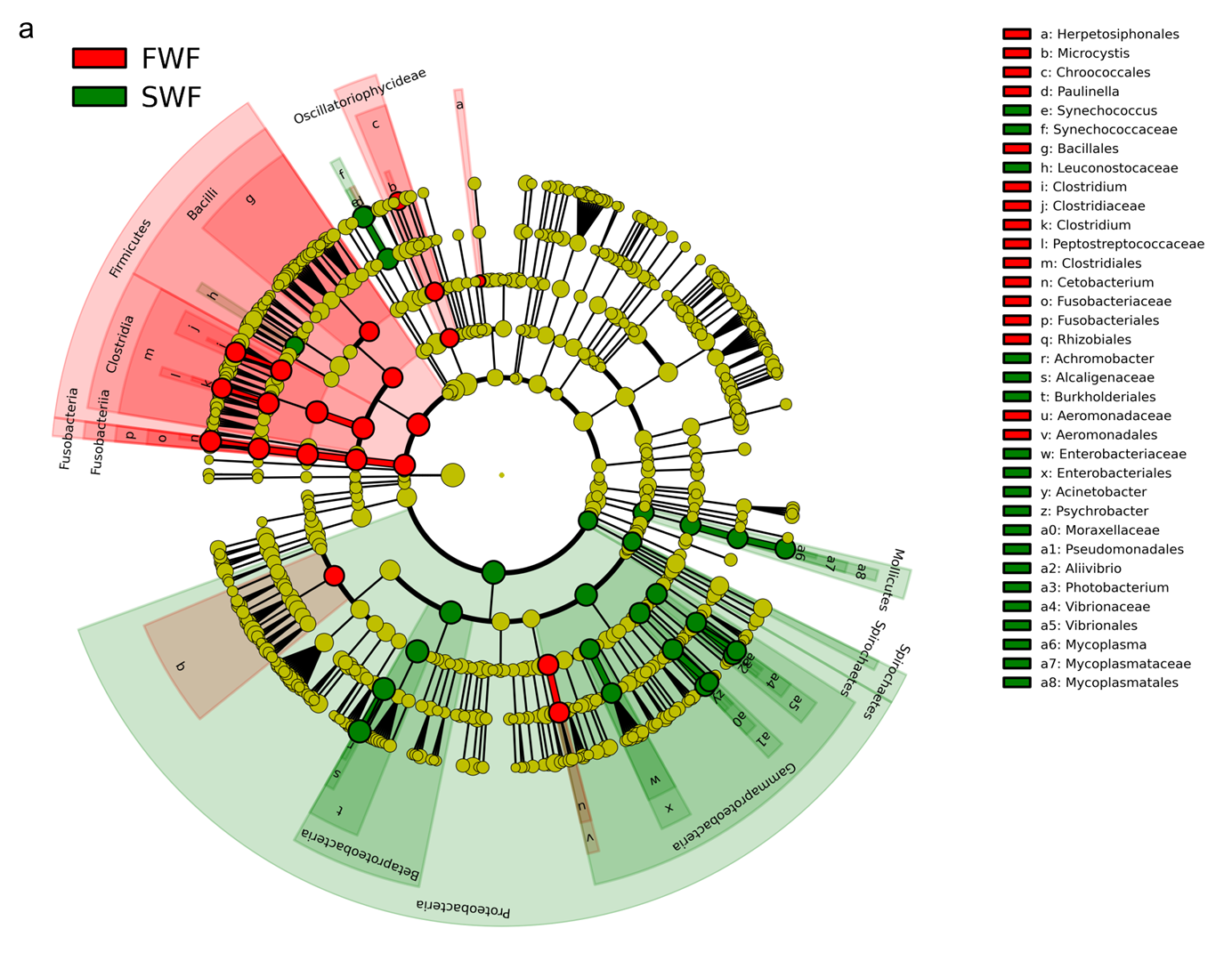

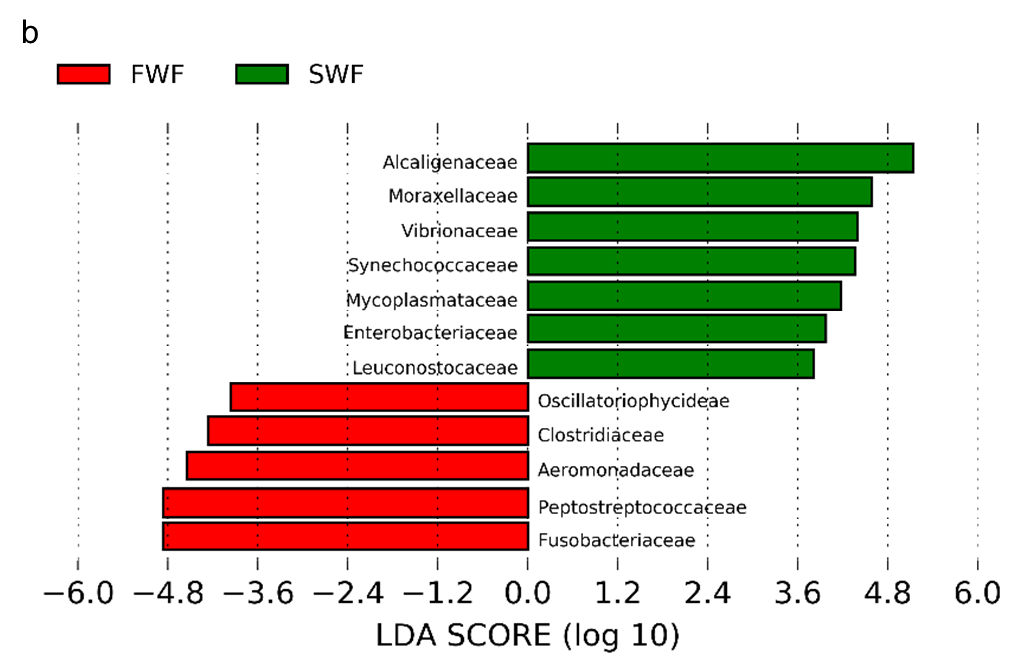


**Supplementary Fig. S6.** Most discriminative bacterial taxa between the gut microbiota of freshwater and seawater fish. (a) Cladogram of bacterial taxa that differ significantly in abundance between freshwater and seawater fish (LDA score > 3.8). (b) Bacterial families that differ significantly in abundance between seawater fish and freshwater fish, as analyzed by LEfSe (LDA score > 3.8).


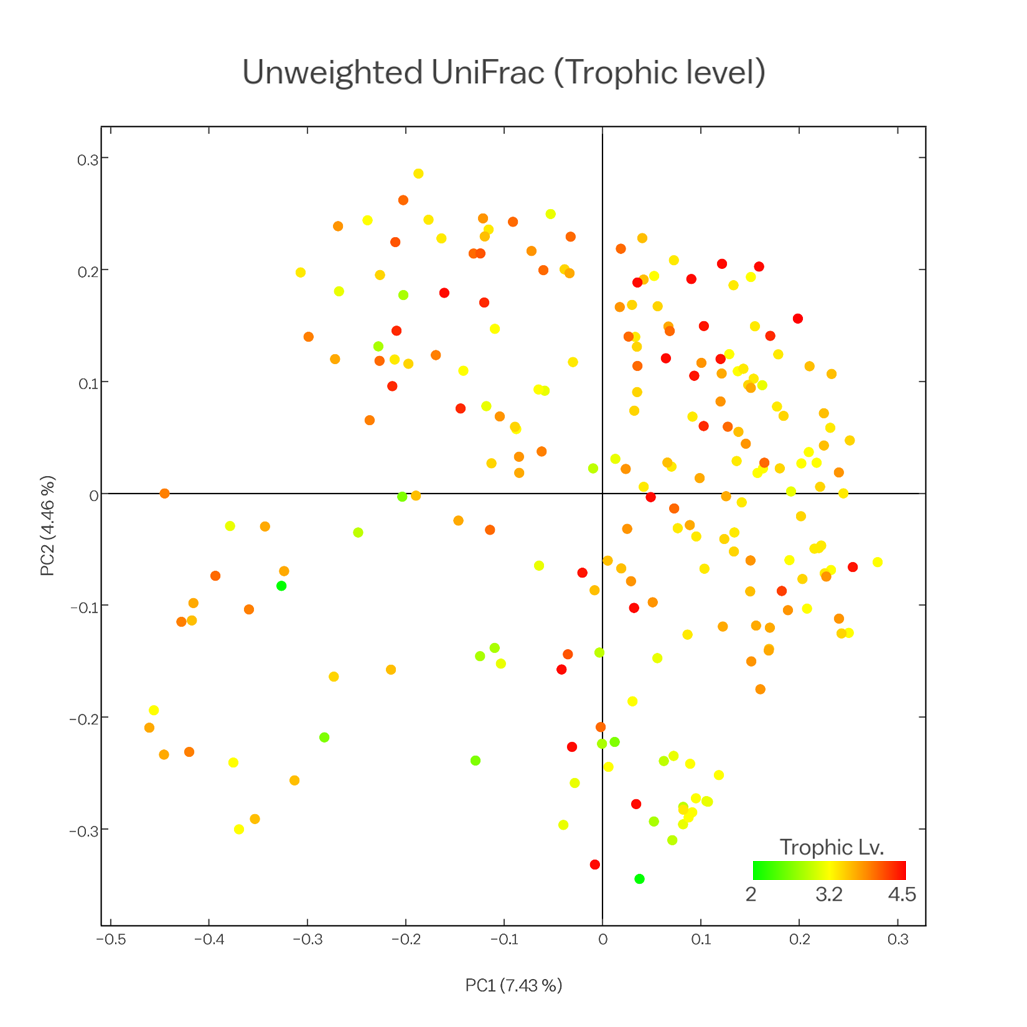


**Supplementary Fig. S7.** PCoA of unweighted UniFrac distances for gut samples from 227 fish. Colors indicate the trophic levels (ANOSIM, *R* = 0.14, p < 0.001).

**
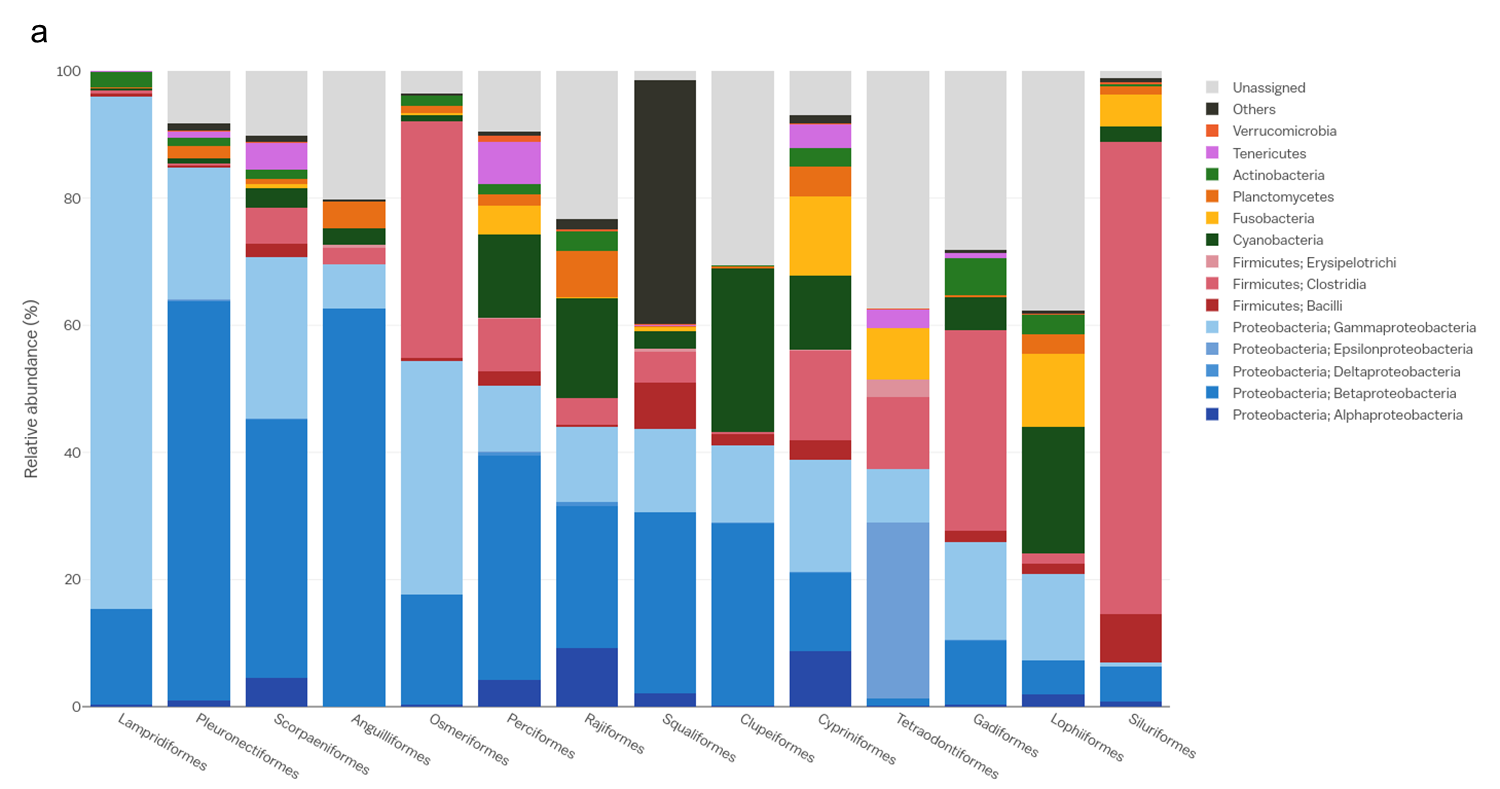

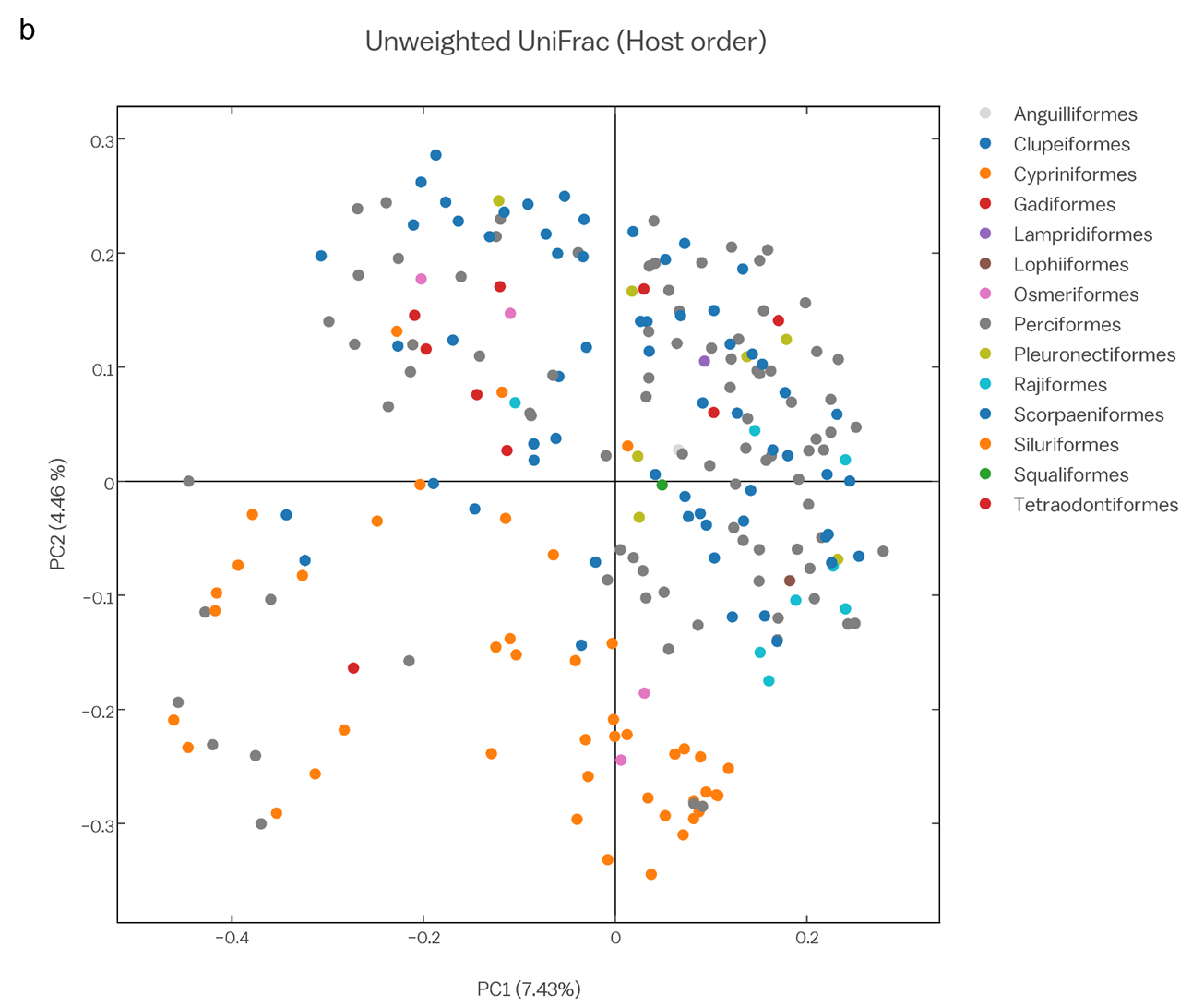
**

**Supplementary Fig. S8.** Gut microbial community composition and structure by fish host order. (a) The mean relative abundance (%) of the bacterial phyla and classes for 14 fish orders. (b) PCoA of unweighted UniFrac distances for gut samples from 227 fish. Colors indicate the host order (ANOSIM, *R* = 0.20, p < 0.001).


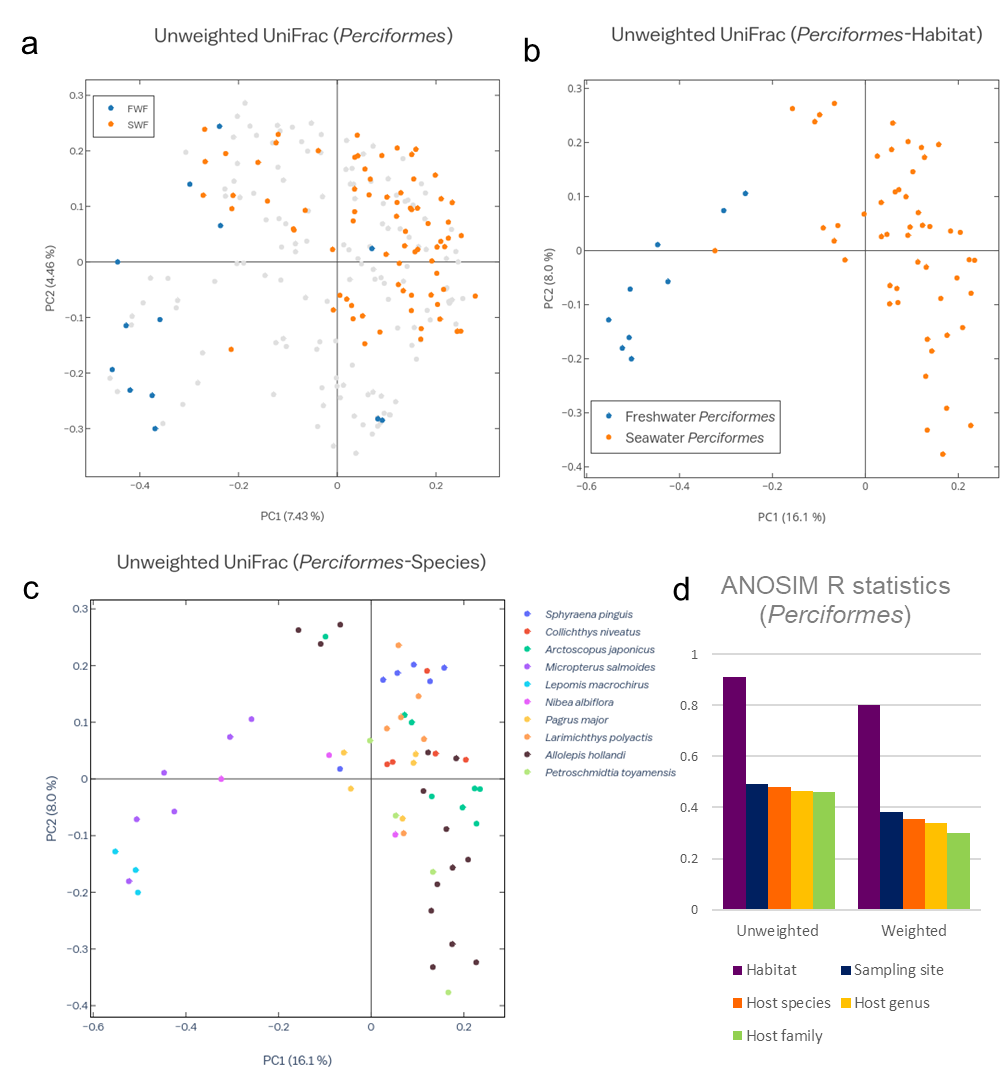


**Supplementary Fig. S9.** Host habitat more strongly determines the fish gut microbiota than host taxonomy. (a) PCoA of unweighted UniFrac distances for gut samples from 227 fish. Freshwater-dwelling Perciformes and seawater-dwelling Perciformes are highlighted for a comparison of an identical host order with different surrounding environments (ANOSIM, *R* = 0.64, p < 0.001). Samples that do not represent Perciformes are marked in grey. (b, c) PCoA of unweighted UniFrac distances for gut samples from 60 Perciformes by habitat (b) and species (c) of each individual. (d) Bar plot illustrating host factors significantly associated with variation in gut microbial taxa. Estimates of variation were derived from between-sample unweighted and weighted UniFrac distances. Size effect and statistical significance were calculated by analysis of similarities (ANOSIM). FWF, freshwater fish; SWF, seawater fish.


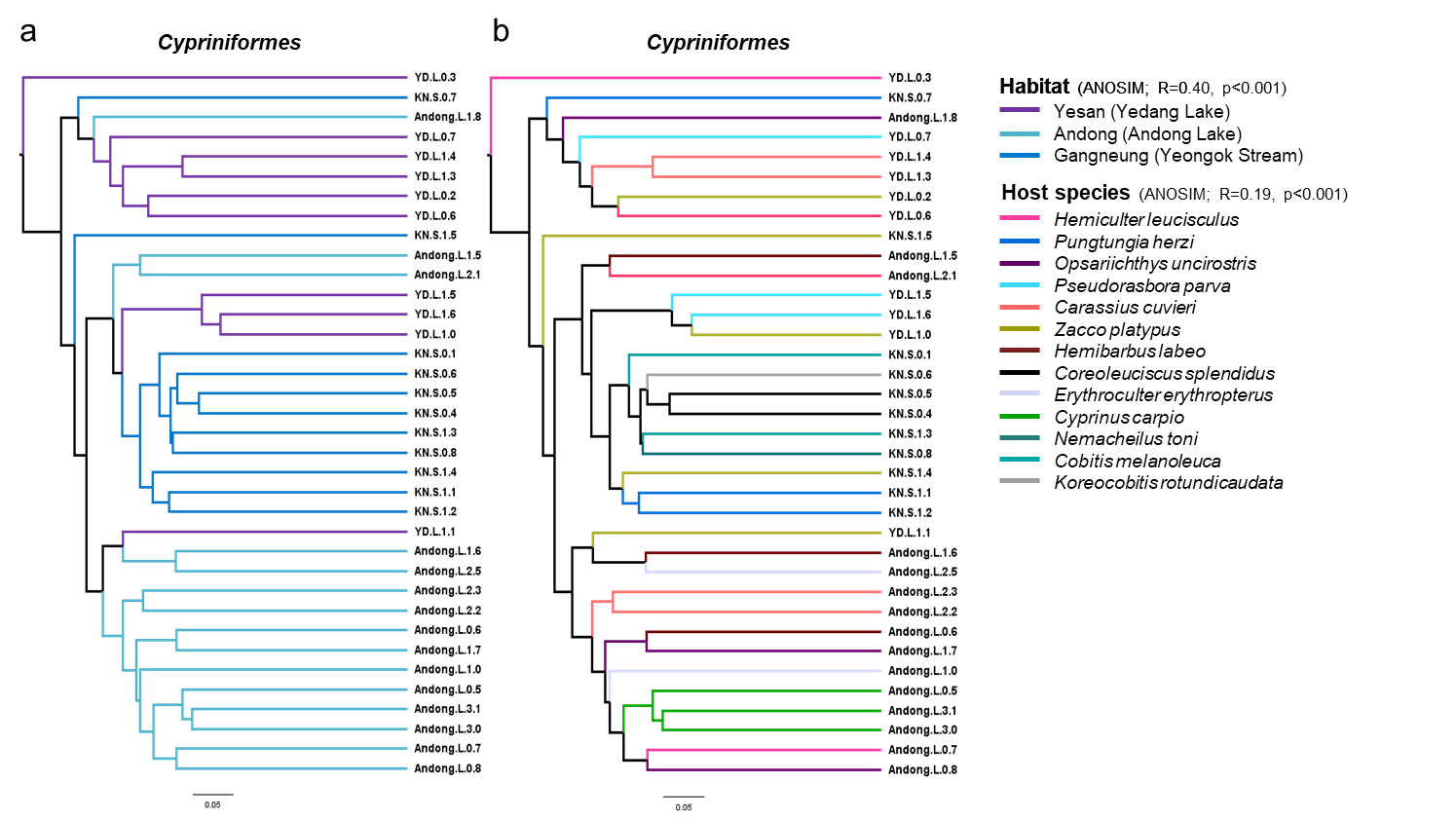


**Supplementary Fig. S10.** Clustering of gut microbial communities in the order Cypriniformes according to habitat (sampling sites) and host species identity. Hierarchical clustering of samples according to unweighted UniFrac distance and dendrograms were constructed using UPGMA, with branches colored according to sampling sites (a) and host species (b).


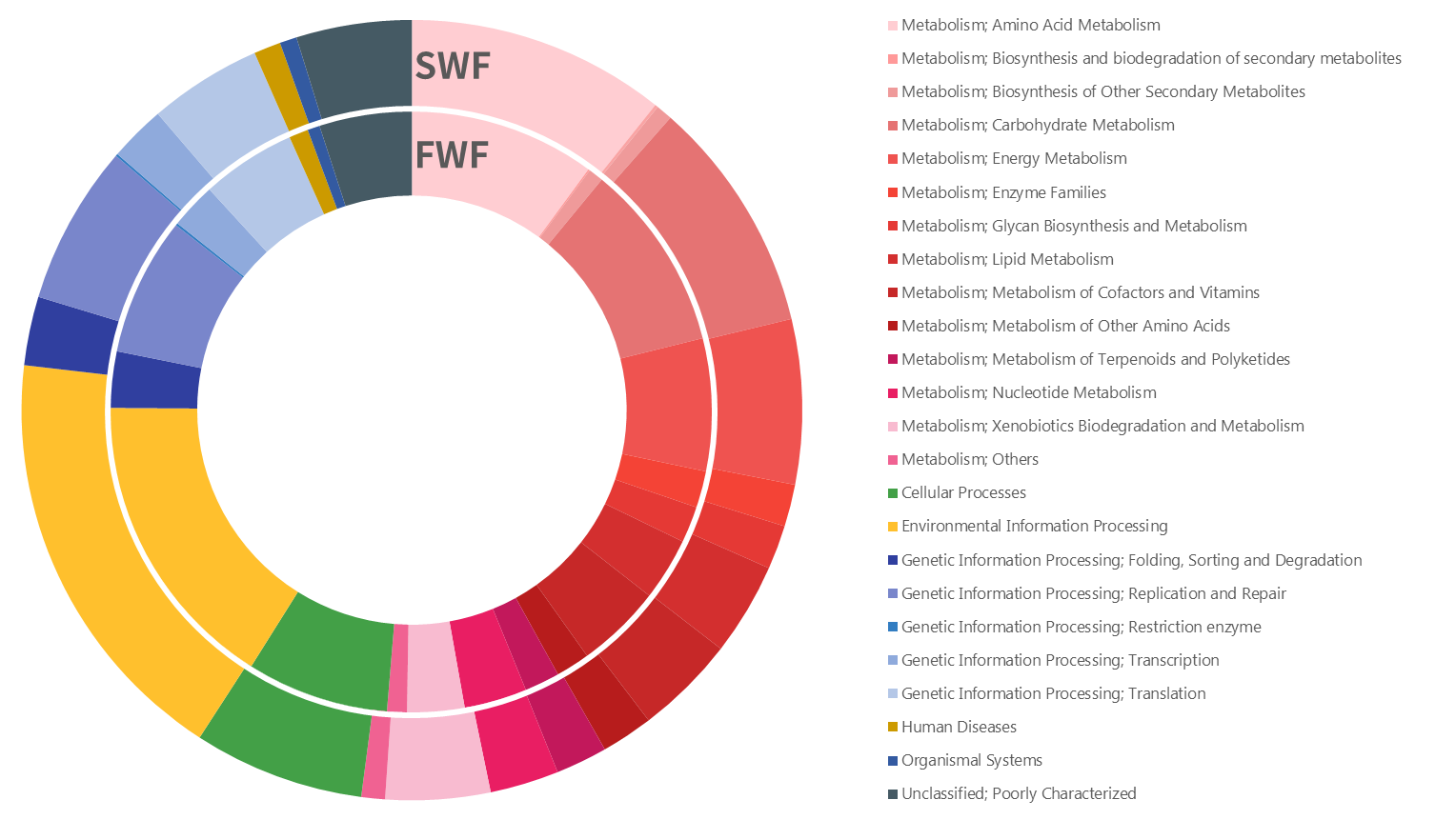


**Supplementary Fig S11.** Predicted functional profiles of freshwater (inner circle) and seawater (outer circle) fish. FWF, freshwater fish; SWF, seawater fish.

**Supplementary Fig S12.** Meta-analysis of fish gut microbiome studies. Top panel: Bray–Curtis dissimilarity-based PCoA using microbial community composition data obtained from various fish gut microbiome studies, including 227 samples from this study, described at the genus taxonomic level. Bottom panel: Boxplots indicate the distribution of values from each fish study along the first principal coordinate. The center line shows the median, the boxes cover the 25th to 75th percentiles, and the whiskers extend to 1.5× the interquartile range. FWF, freshwater fish; SWF, seawater fish.


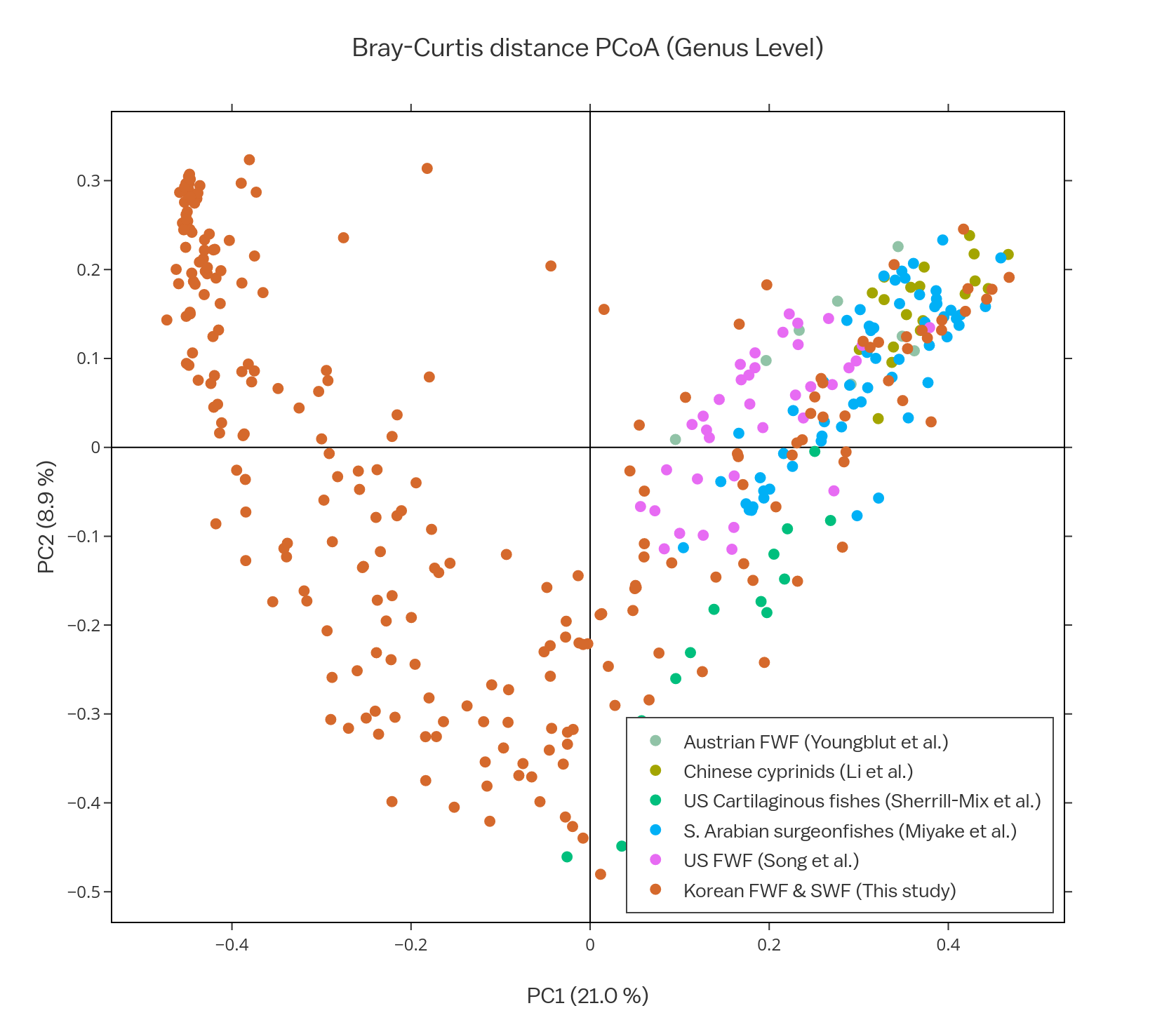

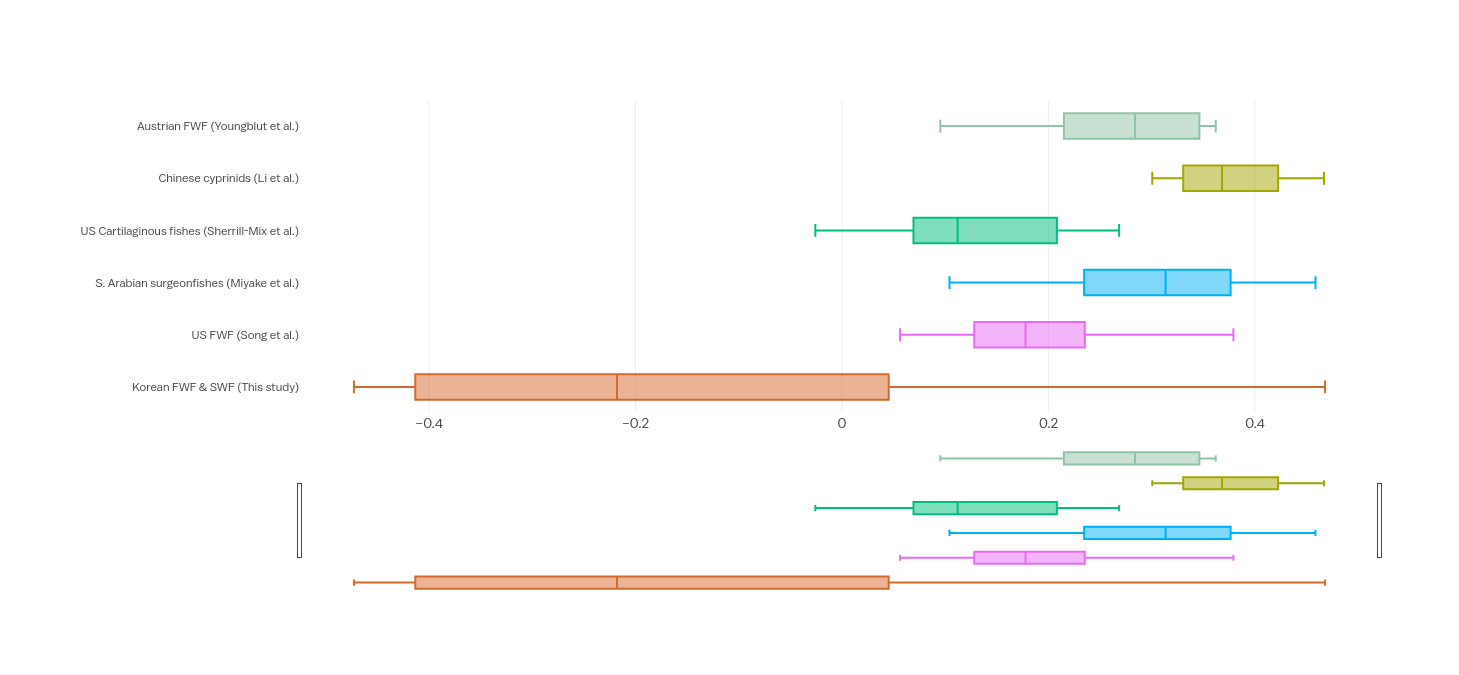

Supplement: Supplementary file 2 — Additional file 1. Supplementary Figures (S1–S12). [file 40168_2021_1113_MOESM2_ESM.docx]
